# Integrated Stochastic Model of DNA Damage Repair by Non-homologous End Joining and p53/p21- Mediated Early Senescence Signalling

**DOI:** 10.1371/journal.pcbi.1004246

**Published:** 2015-05-28

**Authors:** David W. P. Dolan, Anze Zupanic, Glyn Nelson, Philip Hall, Satomi Miwa, Thomas B. L. Kirkwood, Daryl P. Shanley

**Affiliations:** 1 School of Biological and Biomedical Biosciences, Durham University, Durham, United Kingdom; 2 Centre for Integrative Systems Biology of Ageing and Nutrition, Newcastle University Institute for Ageing, Newcastle University, Newcastle upon Tyne, United Kingdom; 3 Eawag—Swiss Federal Institute of Aquatic Science and Technology, Dübendorf, Switzerland; McGill University, CANADA

## Abstract

Unrepaired or inaccurately repaired DNA damage can lead to a range of cell fates, such as apoptosis, cellular senescence or cancer, depending on the efficiency and accuracy of DNA damage repair and on the downstream DNA damage signalling. DNA damage repair and signalling have been studied and modelled in detail separately, but it is not yet clear how they integrate with one another to control cell fate. In this study, we have created an integrated stochastic model of DNA damage repair by non-homologous end joining and of gamma irradiation-induced cellular senescence in human cells that are not apoptosis-prone. The integrated model successfully explains the changes that occur in the dynamics of DNA damage repair after irradiation. Simulations of p53/p21 dynamics after irradiation agree well with previously published experimental studies, further validating the model. Additionally, the model predicts, and we offer some experimental support, that low-dose fractionated irradiation of cells leads to temporal patterns in p53/p21 that lead to significant cellular senescence. The integrated model is valuable for studying the processes of DNA damage induced cell fate and predicting the effectiveness of DNA damage related medical interventions at the cellular level.

## Author Summary

All cells are subject to damage and DNA is the most important molecule to protect. Cells communicate DNA damage through p53—‘the guardian of the genome’—and the dynamics of p53 signalling is one the main mechanisms that determine the outcome for the cell. On detection of DNA damage, p53 is activated and cell cycle arrest is induced: if the DNA damage is repaired quickly then the signalling ends and the cell returns to normal function; if the DNA damage persists then the signalling continues and cells may undergo senescence or apoptosis. Here, we develop a computational model that can simulate the whole process of DNA damage occurrence, DNA damage repair, p53 signalling and cell fate and successfully predict how persistent DNA damage can lead to cellular senescence. The model predicts that using repeating low dose irradiation as a source of damage is as effective as a single large dose, which could have important implications for radiation therapy.

## Introduction

Multiple DNA lesions arise in each cell within an organism every day, caused by errors in DNA replication, by exposure to external factors such as UV light and by a variety of hydrolytic and oxidation reactions [1]. Most simple lesions are repaired quickly and accurately by the cellular DNA-damage response (DDR). The more complex double-strand breaks (DSBs), however, are often left either unrepaired or are repaired incorrectly. Accumulation of persistent DNA lesions leads to apoptosis, cellular senescence or cancer [2,3]. The outcome for a cell after a DNA-damaging insult depends largely on the cell type (or state) and on its DDR capacity: e.g., while irradiation of human fibroblasts in culture leads to cellular senescence [4], irradiating cancer cells leads to apoptosis or mitotic catastrophe [5]. Therefore, clear understanding of control of DDR is important when seeking to identify novel targets for interventions in cancer and ageing [6–9].

Although DNA damage drives cell fate decisions, the actual outcome depends on effects that play out through downstream DNA-damage signalling pathways such as those involving ATM, p53 and p16 [10,11]. Recent studies in ATM/p53 signalling have shown that although the amplitude of the signal is affected by the level of damage, it is the temporal pattern of ATM/p53 activity that more strongly affects cell fate [12,13]. UV-induced damage causes a sustained response of p53 and strong induction of its target p21, leading to senescence, whereas γ-irradiation generates pulses of p53 activity that must endure over time if they are to induce p21 signalling and senescence. Interestingly, regardless of the type of damage insult and the temporal pattern of p53, induction of p21 occurs only in the presence of DNA damage, and not after spontaneous pulses of p53 that occur without damage [14]. Thus, it seems that studying DNA damage signalling without DNA damage occurrence/repair, or vice versa, can explain only part of the cell fate story. A complete explanation requires an integrative, systems-biology approach.

While many separate mathematical models of DNA damage repair and DNA damage signalling exist [15–21], including some from our group, there have been few integrative efforts. Some work has focused on the onset of senescence as a result of damage with varying levels of mechanistic detail [22] [23]; apoptosis has also been included as an alternative cell fate [23]. To date the majority of models are deterministic and DNA damage is considered as a constant input rather than integral part of the system that can change. Two notable integrative studies were done by Passos et al and Ma et al [4,24]. Ma et al. built a model of random DNA damage induction and stochastic repair, ATM signalling and p53/MDM2 negative feedback to explain undamped oscillations in p53 after irradiation. Passos et al added p21-based early senescence signalling downstream of p53 but did not include details of DNA damage repair; their model and accompanying in vitro experiments demonstrated that irradiation-induced senescence requires a positive feedback between reactive oxygen species and DNA damage [4]. Crucially, since their model did not include mechanistic details of DNA damage repair or feedback between p53/p21 signalling and DNA damage factors, it fell short of predicting the amount of senescence after different amplitudes/time courses of irradiation or explaining the long-term decrease in DNA damage repair following irradiation observed in experiments [4,16]. In parallel, a mechanistic stochastic model of DNA damage repair by NHEJ, was developed to try to explain this decrease [16]. This model agreed with the measured repair dynamics for the first eight hours after irradiation (short-term), but overestimated the speed of repair after the eight hour mark (long-term). It was hypothesized that the disparity might be explained by events downstream of the DDR, e.g. cellular senescence.

In the present work we undertake the important task of integrating the model of early senescence with the mechanistic model of NHEJ and examine whether the combined model can both explain the long-term DNA damage repair dynamics and predict senescence for different temporal patterns of irradiation [4,16]. The integrated model was built in rule-based format in BioNetGen [25–27] and for simulation we used the network-free simulator NFsim [28], which lead to a considerable reduction in computation time. We show that the model is able to explain the long-term decrease in DNA damage repair and also to predict experimental results of two p53 signalling studies not used in the model calibration. Additionally, we demonstrate that the model generates a novel prediction that significant cellular senescence can occur after repetitive low-dose irradiation, for which we provide some experimental support.

## Results

### Model construction and overview

**Fig 1:**
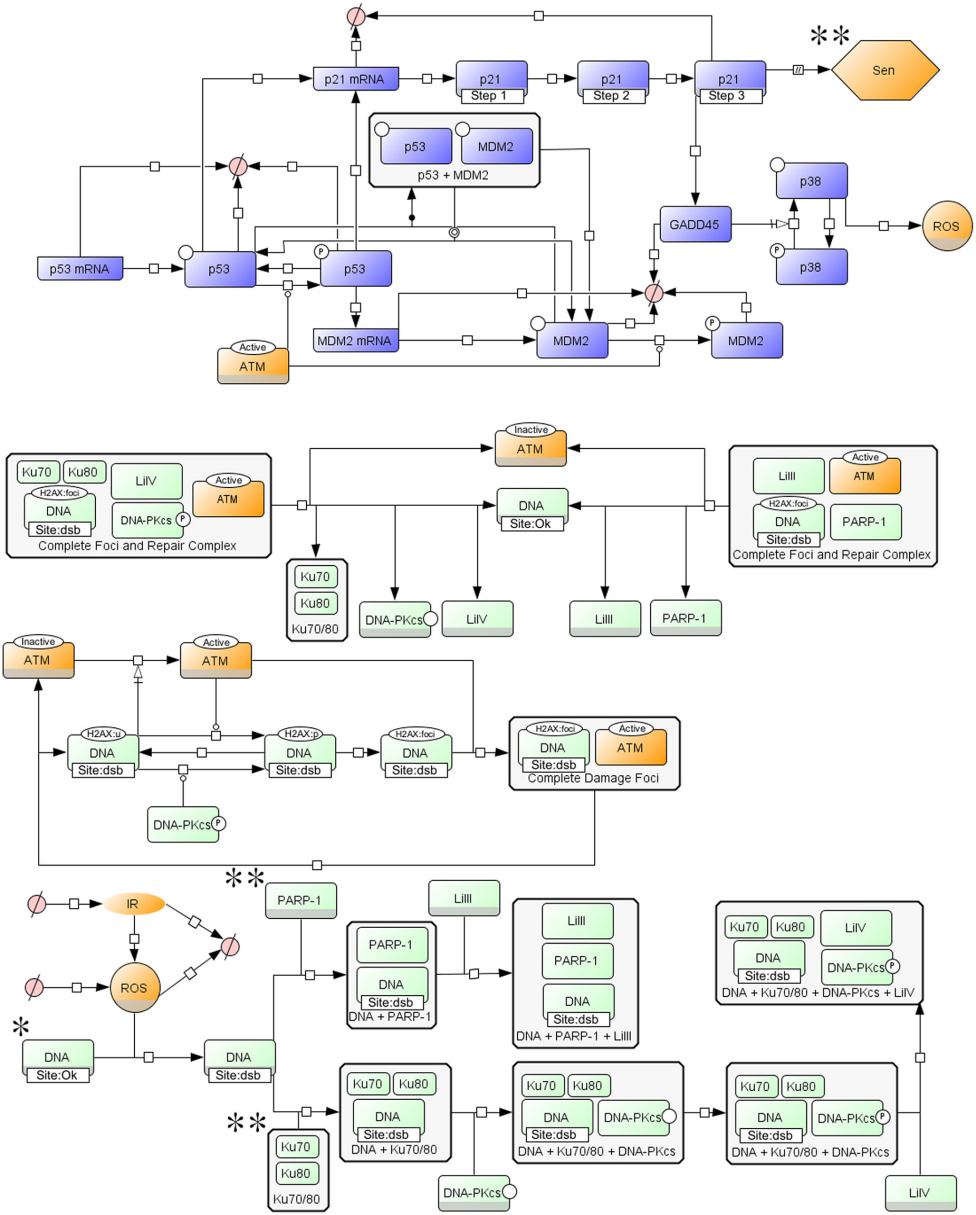
Integrated model of DNA damage repair by non-homologous end joining and p53/p21 mediated early senescence signalling. A basic graphical model in SBGN process diagram format [58], with NHEJ in green [16], p53/p21-mediated early senescence signalling in blue [4] and parts of the model that have been recalibrated or added in orange. In the NHEJ part of the model, DNA double stranded breaks are induced and then repaired by either D-NHEJ or B-NHEJ. In the signalling part, activated ATM triggers phosphorylation of the p53/p21 and activation of pathways that lead to senescence. The DNA entities (marked by *) represent 50 separate DNA species that undergo damage and repair independently. After the cells reaches an early senescent state (Sen) the abundance of Parp-1 and Ku70/80 slowly decreases (**) (see S2 Table for details). The presented NHEJ part is a reduced version of the full NHEJ part with two types of double-stranded breaks (simple and complex) (see S1 Text for detailed description of the model). A gray bar at the bottom of a molecular species indicates that the same species is presented several times in the model; in other words the same molecule can participate in many different reactions. A full description of SBGN, and a key for the components in an SBGN compliant process diagram can be found at http://www.sbgn.org/

We built a single-cell stochastic model of non-homologous end joining (NHEJ), DNA damage signalling and early senescence signalling by developing and integrating two recent stochastic models: one of DNA damage repair by NHEJ [16] and one of irradiation-induced senescence focusing on the DDR [4] (Fig 1). The model describes the formation of DNA DSBs by irradiation and background ROS and their repair via the fast DNA-PKcs NHEJ (D-NHEJ) and the slower backup NHEJ pathway (B-NHEJ), also known as microhomology-mediated end joining [29]. In D-NHEJ, Ku70/80 and DNA-PKcs are recruited to the DSB and form a complex. Ku80 is redox sensitive, which causes an increase in the rate of Ku70/80 dissociation from a DSB with high ROS levels [30]. DNA-PKcs then undergoes autophosphorylation and ligase IV (LiIV) ligates the DSB. In B-NHEJ, Parp-1 is recruited to the break site followed by ligase III (LiIII) which ligates the DSB [31,32]. Since tracking the dynamics of individual DSB breaks was one of the aims of the study, each DNA break site was modelled as an independent species.

In addition to binding of Ku70/80 and Parp-1, the DSB causes activation of ATM which phosphorylates H2AX leading to formation of a DNA damage focus [33]. Each focus resolves independently of the DSB repair (i.e., a focus can be resolved even if the DSB is still present and in that case a new focus often forms, but it is also possible for the focus to still exist after the DSB has been repaired), but the repair of the DSB requires the presence of the damage focus [34]. The phosphorylation of H2AX at the site of a DSB can also be carried out by the other Phosphatidylinositol 3-kinase-related kinases (PIKK) present in the model DNA-PKcs [35] once it has undergone autphosphorylation. Activated ATM phosphorylates p53, which increases its stability by reducing its capacity to bind to MDM2 for targeted degradation. Activated p53 increases transcription of MDM2 and CDKN1A (p21). p21 in turn activates early senescent signalling (phenotype Sen in Fig 1), increasing production of ROS via GADD45 and phosphorylation of p38. Once early senescent signalling is activated, the repair factors Ku70/80and Parp-1 are depleted to the levels observed in senescent cells [36,37] (S2 Table).

Our model of DNA damage repair is limited to NHEJ as this is the dominant mechanism of repair during the G1 phase of the cell cycle [38] which also corresponds to the state of cell cycle arrest for early senescent cells. G1 lasts approximately 24 hours in MRC5 human fibroblasts (unpublished observation), and we assume the validity of the model for early senescent cells for at least this period of time. As the senescent state deepens other processes take over [39,40] and the validity of the model prediction much beyond 48 hours has to be regarded with some caution.

The original, separate models were created in the SBML format; however, due to their size, SBML was unsuitable for their integration. Instead, the original reactions were translated to a BioNetGen rule-based format [25,26] with appropriate recalibration where the separate models overlapped (Fig 1 and Methods). The integrated model, consisting of 4,583 species and 22,341 reactions, is available to download as a bngl file in Supporting Information. A full description of the model reactions and reaction rates can be found in the Supplementary Information (S1 Text and S1–S3 Tables).

### Model parameterisation and validation

In all key readouts, the integrated model matched qualitatively the output of the original published models. To ensure the consistency of the integrated model, and to constrain further its parameters, we used three additional published datasets that were not used in constructing the original models [13,16,41]. For each dataset a subset of the model parameters was recalibrated, while ensuring that the results matched the dataset used in the construction of the original models. These recalibrated parameters were: 1) the rate at which p21 activity leads to early senescence, established by Passos et al and based on measurement of senescence after a single level of irradiation (selected because the model is very sensitive to its value) [4]; and 2) the parameters of the feedback between senescence and abundance of Ku70/80 and Parp-1, as literature only provided approximate steady-state levels of both molecules after senescence and not the dynamics of their change [36,42] (S2 and S3 Tables). A further parameter that was altered from the original models was the rate of background ROS production, which was determined experimentally (S3 Table).

**Fig 2:**
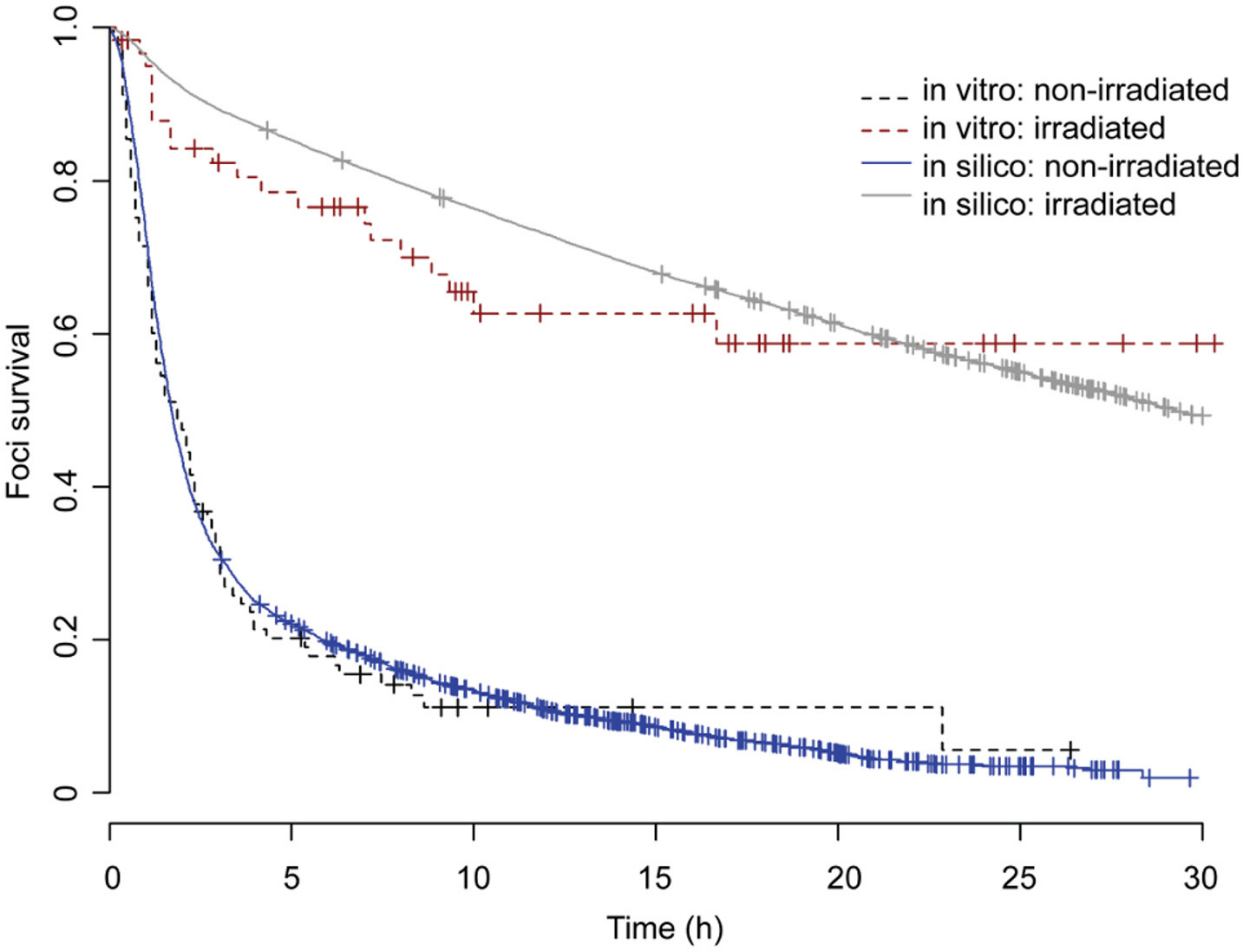
DNA damage foci longevity. Simulated (solid) and experimental (dotted) DNA damage foci survival curves are not statistically significant for both irradiated cells (p = 0.315, log rank test) and non-irradiated cells (p = 0.65, log rank test) (part of the experimental data has already been reported in [16]). n ≥ 100 cells per experiment and n = 120 simulations.

The first dataset used was the full dynamics of DNA damage foci observed in MRC5 fibroblasts after irradiation [16]. Whereas only short-term data was used in parameterization of the original NHEJ model, we here included the long-term data. As noted earlier, the original NHEJ model was able to explain short-term but not the long-term dynamics of DNA damage foci [16]. The early-senescence feedback, which increases the production of ROS and depletes the repair factors Ku70/80and Parp-1, enabled the model to match both short-term and long-term dynamics of DNA damage foci (Fig 2 and S1 Fig). Resolution of foci is faster in non-irradiated cells compared to irradiated ones; additionally almost all foci in non-irradiated cells resolve in 24 hours, whereas only around half of the foci in irradiated cells do so. All in all, the decrease in DNA damage repair after irradiation can be explained by a reduction in the efficiency of D-NHEJ and a shift from the D-NHEJ in low ROS normal cells to the slower B-NHEJ in high ROS/senescent cells, as has been demonstrated in the original model [16], and by a stabilization of high ROS levels and further reduction of both D-NHEJ and B-NHEJ due to depletion of Ku70/80 and Parp-1 after early-senescence signalling has become activated, which was added in the integrated model. The proposed early senescent signalling feedback is therefore a plausible mechanism behind the long-term decrease in DNA damage repair.

**Fig 3:**
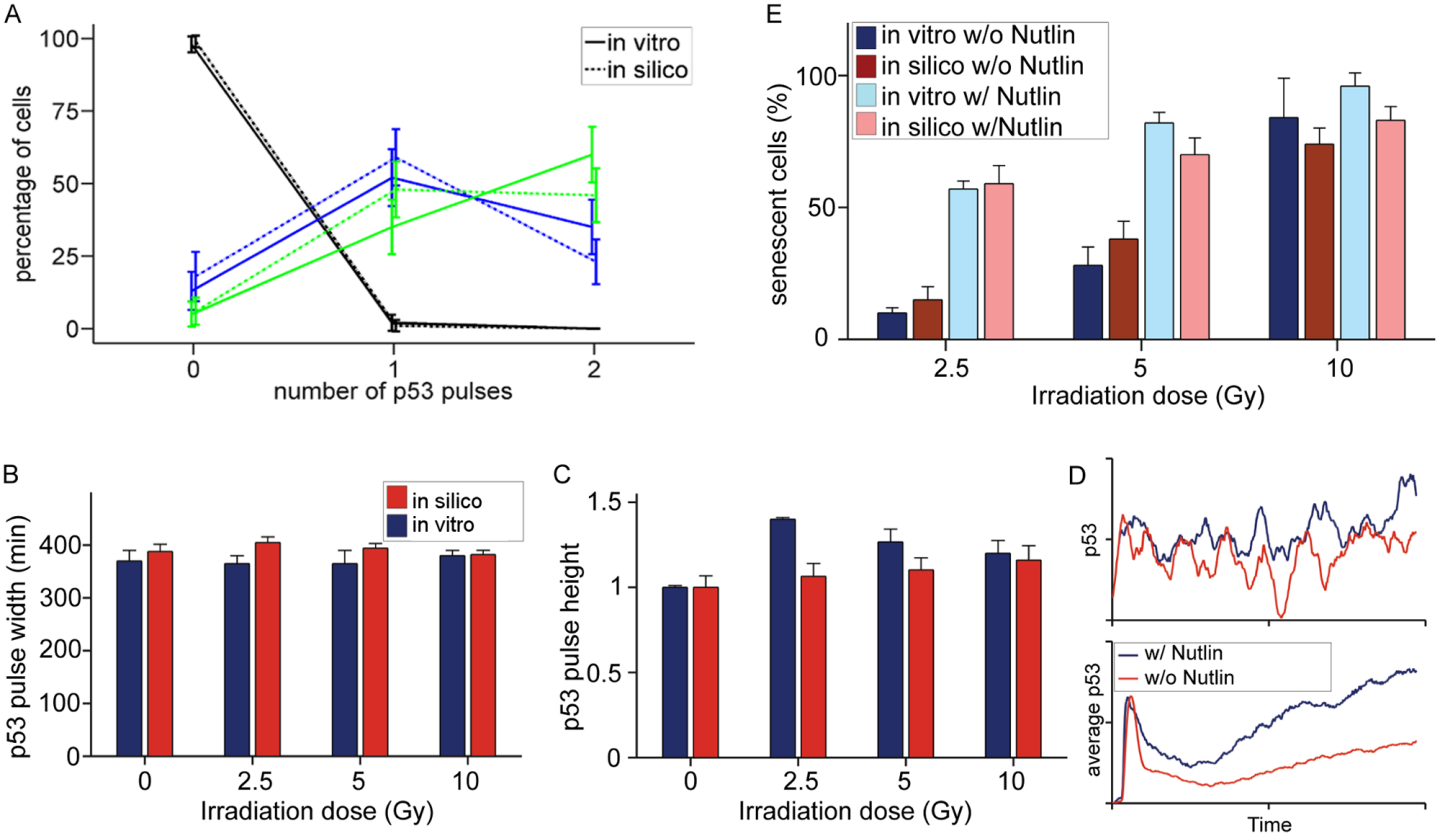
p53 signalling and senescence after irradiation. Comparison between single cell measurements of p53 dynamics after irradiation and integrated model simulations (experimental data from [41]) for A) number of p53 pulses observed in the first 16 h after irradiation with 0 Gy (black), 2.5 Gy (blue) or 10 Gy (green). B) pulse width and C) normalized pulse amplitude. D) p53 response to 5 Gy irradiation with and without Nutlin-3 inhibition; representative single runs (top), population average (bottom). E) Measurement [13] and simulation of senescence after irradiation with or without Nutlin-3 inhibition. Data are presented as mean ± 95% confidence interval (A,D) or mean ± SE (B,C). n ≥ 100 cells per experiment [13] and n ≈ 200 simulations.

The second dataset used was from a study that measured the dynamics of p53 after exposing MCF7 breast cancer cells to different levels of irradiation [41]. The same dataset was also used for validation purposes by Ma et al [24]. The study showed that the number of p53 pulses depends strongly on the level of irradiation, but the average amplitude of the pulses and time between consecutive pulses have only a weak dependence, a phenomenon they named “digital behaviour of p53 oscillations”. The population data derived from a minimum of 100 cell simulations agreed very well with the measured p53 pulse properties (Fig 3A, 3B, and 3C) (t-test; p > 0.05 for all experiment vs simulation comparisons, except pulse amplitude at 2.5 Gy, where p < 0.001). The discrepancy in p53 pulse amplitude at 2.5 Gy (Fig 3C) may be attributed partly to the stochasticity of the system, but may also arise from the lack of a precise definition of a pulse in Lahav et al (for the definition of pulse used in this study see S2 Text). In accordance to previous observations, the p53 amplitude was much more variable (coefficient of variation of about 60%) than the period between pulses (coefficient of variation about 25%)[43]. The width of each p53 pulse was 350 ± 160 min in Lahav et al. vs 385 ± 95 min predicted by the model. The time to the first pulse peak was 360 ± 240 min vs 355 ± 165 min, and the time between consecutive pulses was 440 ± 100 min vs 495 ± 130 min. These results computationally confirm that the link between DNA damage repair and p53 signalling is well represented in the integrated model.

The final dataset used was from a recent study where MCF7 cells were exposed to different levels of irradiation whilst inhibiting the p53-MDM2 interaction with Nutlin-3 [13]. The p53 abundance was measured immediately after irradiation and the number of senescent cells several days later. Using the same time course concentration of Nutlin-3, the p53 response in simulations of the integrated model switched from pulsed to a more sustained p53 signalling (Fig 3D). Although single simulations of the p53 response to irradiation with Nutlin-3 inhibition did not produce a consistently high signal, the levels of p53 did not fluctuate nearly as much as without Nutlin-3 inhibition and were on average much higher. At the same time, after recalibrating the rate at which p21 leads to early senescence (MCF7 cells were more robust than the MRC5 cells used for calibrating the original models) the fraction of cells entering early senescence in the simulations matched well with the fraction of Sen-β gal stained cells in the experiments [13](Fig 3E; Chi-square, p > 0.05 for all experiment vs simulation comparisons), again suggesting that the abstracted presentation of senescence in our model is adequate.

### Stochastic simulations of cell fate

It has been shown that different patterns of p21 after irradiation can explain why some cells progress to senescence while others escape [4]. However, the contributions of DNA damage repair and signalling to senescence have not yet been analyzed. We reasoned that looking into individual stochastic runs of the recalibrated model could provide valuable insight.

**Fig 4:**
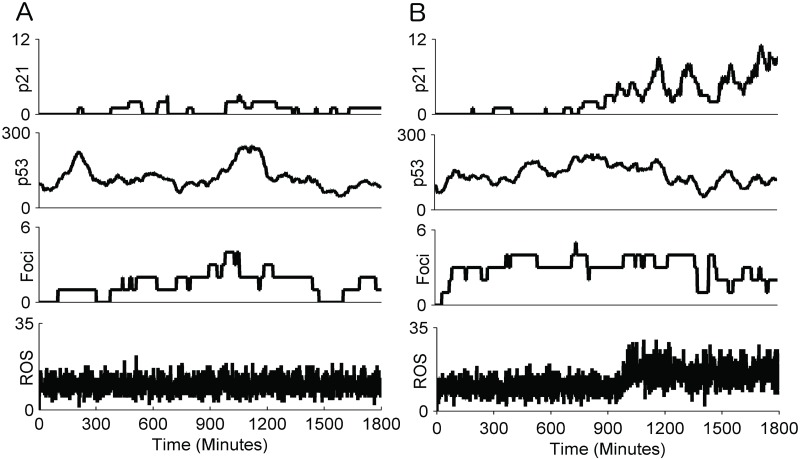
Single cell simulations for non-irradiated cells. The time courses for number of molecules of ROS, DNA damage foci, p53 and p21 for two representative simulations of the integrated model with normal background ROS (S3 Fig): A) no p21/ROS feedback activated, B) p21/ROS feedback activated (rare event under normal conditions).

In non-irradiated cells under normal conditions the abundance of ROS is approximately constant. The actual number of DNA damage foci in a cell at any time varies stochastically, a higher number of ROS molecules usually but not always producing more DSBs. The induction of DNA damage subsequently increases p53, which, due to the feedback between p53 and MDM2, becomes a transient pulse [44]. Higher levels of p53 lead to an increase of p21, which in turn leads to additional production of ROS, providing the p21 levels are sufficiently high (compare Fig 4A and 4B). Fig 4B shows how a relatively high maintained number of DNA damage foci results in continuous activation of p53, which eventually increases p21 in the cell, ultimately increasing the level of ROS. In non-irradiated cells the repair machinery is able to keep the levels of damage under control; none of the simulated cells progressed into early senescence under normal background levels of ROS. However, if the background ROS production was increased 10-fold, the number of induced DNA damage foci increased and 32% of cells entered into early senescence after 30 hours.

**Fig 5:**
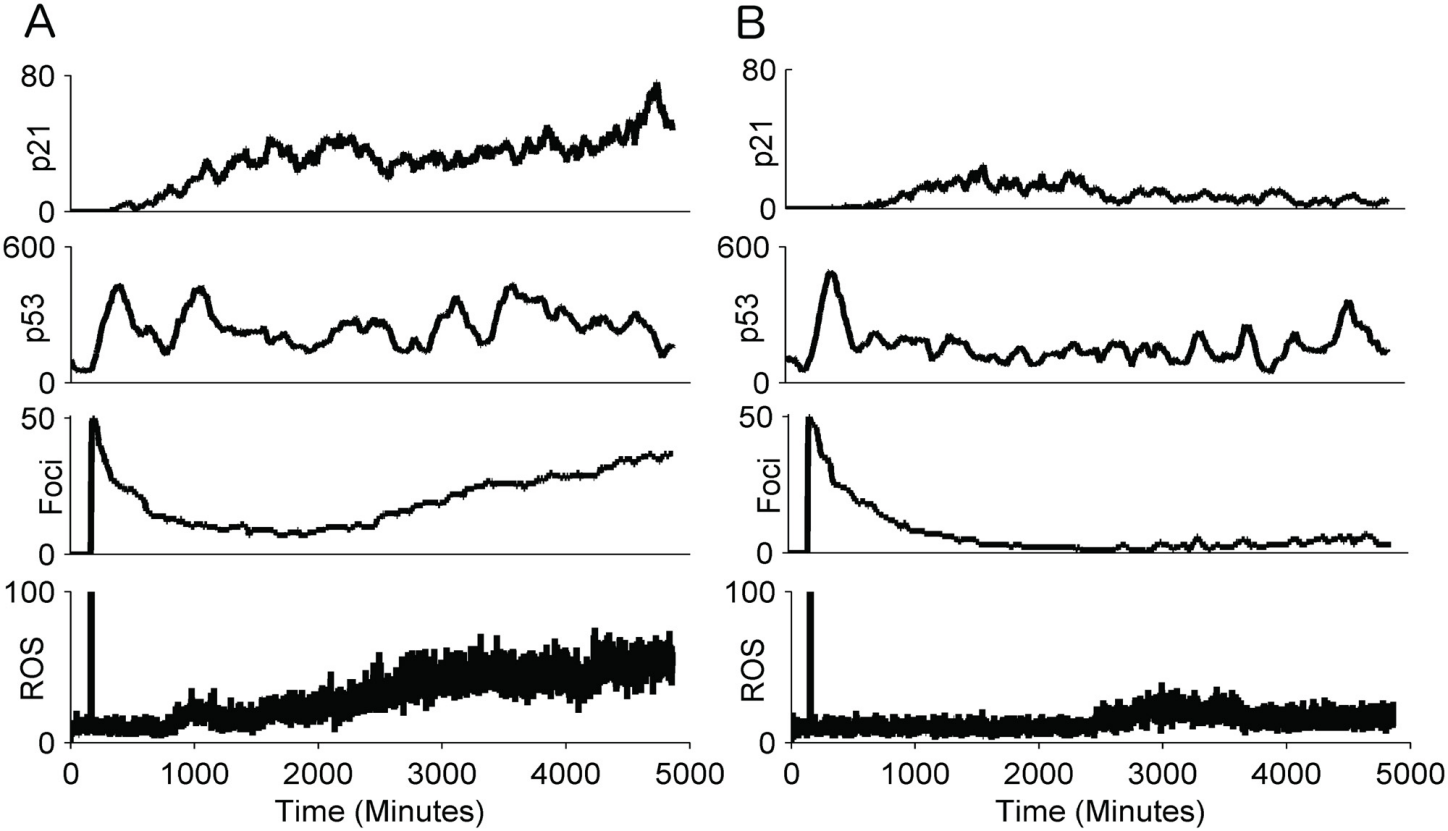
Single cell simulations for irradiated cells. Time courses for ROS, DNA damage foci, p53 and p21 for two representative simulation of the integrated model after a 20 Gy irradiation event: A) cell that entered early senescence, B) cell that escaped early senescence.

Simulations showed that cells have much more difficulty coping with high-damage events; in our case this was the 20 Gy X-ray typically used to generate irradiation-induced senescence. A pulse of irradiation induces a large number of DNA damage foci which are rapidly repaired through D-NHEJ and B-NHEJ (Fig 5). The p53 levels peak about 6 hours after irradiation (Fig 5 and S2 Fig). If repair is rapid the level of DNA damage decreases enough to prevent a next high pulse of p53 (Fig 5B), which in turn prevents p21 from causing early senescence and from feeding strongly back into ROS production.

In the simulations, over three and a half days approximately 85% of all cells progressed into early senescence, while 15% did not. Of these 85%, almost all had a second large (but generally smaller than the first) peak or a sustained high level of p53 shortly after the first peak (Fig 5A), consistent with previous studies [12,41]. In the early-senescent cells the ROS levels and the number of DNA damage foci rose, and there were repeated pulses of high p53 and an increasing p21 signal. The average signals show an increase with time for ROS, DNA damage foci, p53 and p21 (S2 Fig), as more cells enter early senescence. If the irradiation pulse is decreased, the feedbacks are weaker and fewer cells enter early senescence over three and a half days (38% for 5 Gy, 74% for 10 Gy, 85% for 20 Gy).

Ultimately, the difference between non-irradiated and irradiated cells is the repair machinery’s capacity to handle the level of damage induced. While the low level of damage in the non-irradiated cells poses little problem, the higher level in cells that have undergone significant amounts of stress is much more likely to overwhelm the repair system. If the damage is not repaired fast enough the feedback reduces the cell’s repair capability, increasing the longevity of DNA damage foci and leading to senescent cells with several permanent DNA damage foci [4].

### Fractionated low-dose irradiation can cause senescence

**Fig 6:**
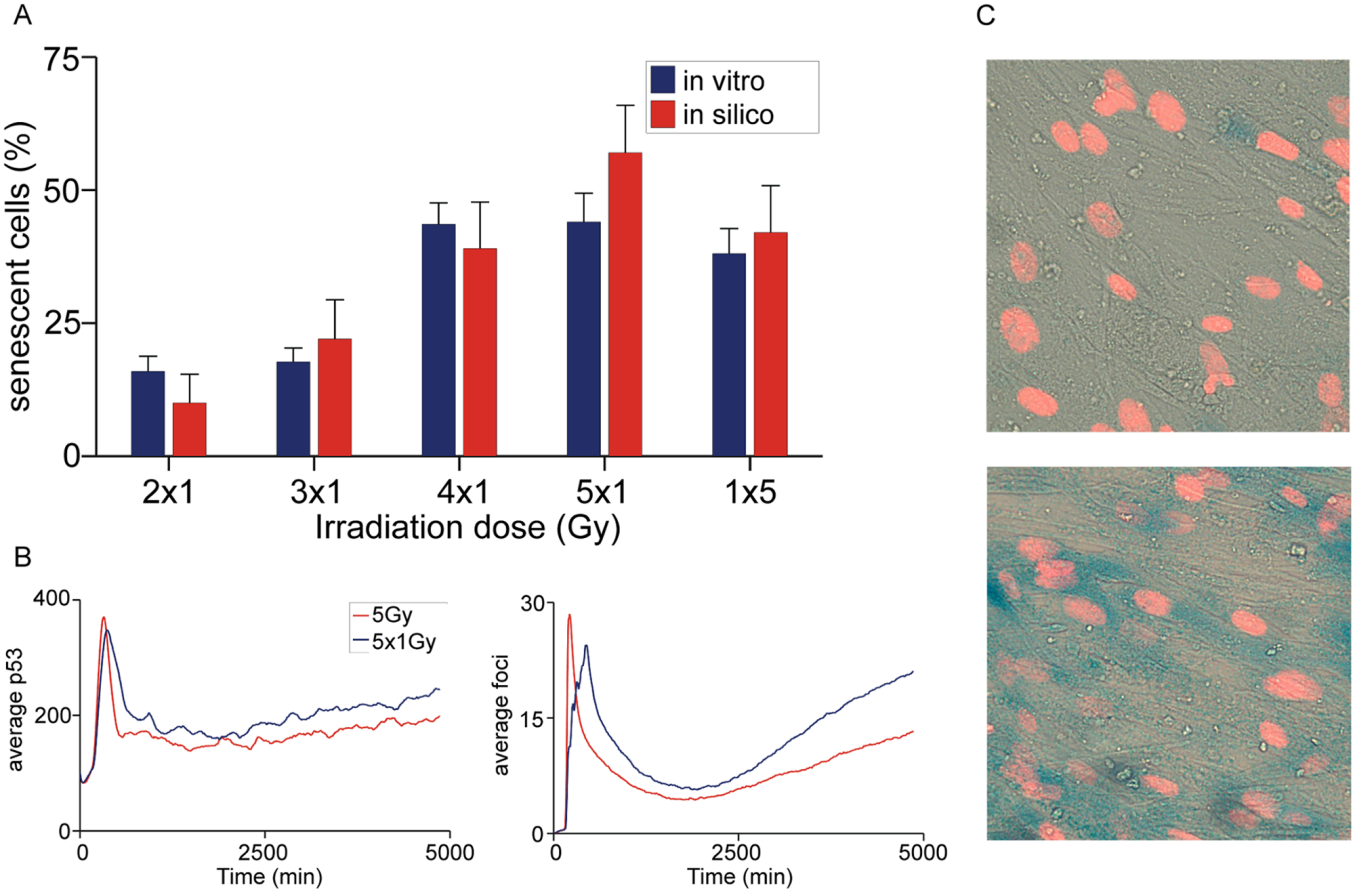
Senescence after fractionated irradiation. A) Fraction of senescent cells for different temporal exposures to irradiation as predicted by the integrated model and measured experimentally. B) Simulated population average time course of DNA damage foci and p53 after 5 x 1 Gy and 1 x 5 Gy irradiation. C) Images of cells stained for senescence after fractionated irradiation with a single 1 Gy pulse (top) and 5 x 1 Gy pulses (bottom). Data are presented as mean ± 95% confidence interval, n ≈ 200 simulations.

The standard protocol for irradiation-induced senescence involves exposing cells to a single 20 Gy pulse of irradiation [4,45]. However, since the temporal pattern of DNA damage seems to have as great an effect on cell fate as the amplitude of the DNA damage signal, we wished to see whether a fractionated exposure to low-dose irradiation was also likely to produce senescence (S3 Fig). Simulations predicted that a single 5 Gy pulse would cause 38% of the cells to undergo early-senescence; however this would be increased to 55% if 5 x 1 Gy pulses were used instead (1 pulse/1 hour or 1 pulse/2 hours) (Fig 6A). The simulations also suggested that each additional 1 Gy pulse would push more cells into senescence than the preceding one, but that senescence would plateau after five pulses. A single 1 Gy pulse would push 3% of cells into senescence, 2 x 1 Gy pulses 10%, 3 x 1Gy pulses 22% and 4 x 1 Gy pulses 39% (Fig 6A). The model does not predict that 5 x 1 Gy irradiation pulses produce more DNA damage breaks than a single 5 Gy pulse, however it predicts a delay in the decrease of p53 levels and thus widening of the first p53 pulse (pulse width 585 ± 170 min) (S4 Fig). This delay is enough to induce senescence in an additional 17% of cells. While both intervals between pulses were predicted to cause about the same increase in senescence, their p53 dynamics were different: 2 hours between pulses caused lower p53 amplitudes but wider pulses, whereas 1 hour between pulses caused higher p53 amplitudes, but a narrower pulse.

The model’s predictions were tested by irradiating MRC5 cells with the same time-course of irradiation as used in the model, with Sen-β gal and cell-cycle arrest staining being used as proxies for senescence (Fig 6C). We opted to investigate the temporal pattern with 1 hour between irradiation pulses as it produced a clear distinction in the production of a senescent population within the model simulations and would take much less time than the 2 hour pattern. The experiments confirmed the model predictions that a series of low-dose pulses produced significant senescence and that the higher the number of pulses the more senescence occurred (Fig 6A). However, the experimental data suggested a plateau in the senescent population already after 4 x 1 Gy pulses, just above the values seen from a 1 x 5 Gy dose, suggesting that the delay in the decrease in p53 level achieved by repeated pulsing did not produce the entire predicted effect. When trying to adjust the model parameters to match this data, we were not able to achieve an improvement by changing any single model parameter, suggesting that either more experimentally driven parameter estimation is necessary or that more details should be included in the model to fully capture the process of DNA damage-induced early senescence signalling.

## Discussion

In this study we constructed an integrated model combining two previous stochastic models, one of DNA damage repair by non-homologous end joining, the other of p53 signalling-induced cellular senescence (Fig 1). We simulated the production and resolution of DSBs in the G1 phase of the cell cycle with and without induction by irradiation. Damage by ROS is the most common source of DSBs even in unirradiated cells although it should be noted that DSBs may also be generated in S-phase which we do not consider here. The integrated model is, to our best knowledge, the first stochastic mathematical model that takes into account both the mechanistic details of DNA damage repair, the downstream DNA damage signalling which leads to cell fate choice and the feedback in-between. The integrated model captures much more of the relevant biology and should therefore provide significantly enhanced explanatory and predictive power.

Recent experimental data shows that irradiation not only induces DSBs, but also decreases the rate of DNA damage repair [16]. The shift towards slower repair occurs during the first minutes/hours after irradiation and does not reverse for cells entering senescence (Fig 2 and S1 Fig). Our integrated model shows that the long-term slowdown can be attributed to the redox sensitivity of Ku70/80, increased production of ROS and decreased transcription of Ku70/80 and Parp-1 during early cellular senescence (Fig 2). Although the slower repair in the model is due primarily to the redox sensitivity of Ku70/80, the higher number of complex DSBs being formed by increased levels of stress, from irradiation or ROS generated as a result of the DDR, also contribute to slowing the rate of observed repair. This result significantly extends the capability of one of the earlier models, which showed that the short-term part of the shift can be explained by redox sensitivity of Ku70/80, but which could not explain the persistence of DNA damage in the long-term [16]. Recently, it has been suggested that a high proportion of permanent damage foci are telomere associated, and thus resistant to NHEJ repair [46,47], and this will need to be included in the model for longer term predictions.

The model simulations also matched the p53 dynamics determined experimentally by single-cell imaging after irradiation. While Ma et al’s model was also able to explain this digital behaviour, it could not match the cell-cell variability found in the experiments [24]. Although other mathematical models, which did not explicitly account for DNA damage or the dynamics of its repair, have been used to explain cell-cell variability of the p53 system after irradiation [43], the deterministic approach used in those models required low-frequency noise to be added to match the experiments. In contrast, our integrated stochastic model was able to match the observed cell to cell variability in p53 signalling without adding additional noise. Slow fluctuations in p53 occurred in cells with little DNA damage, a smaller number of pulses in cells where DNA damage was successfully repaired and continuing pulsing in cases where DNA damage was not repaired (eventually leading to senescence) (Fig 5). Furthermore, our predictions on the entrance of cells into cellular senescence, which greatly depends on the p53/p21 signalling, also matched the experimental data (Fig 3) [13].

Following the proposal of Lahav et al that the temporal pattern of p53 signalling plays a decisive role in determining cell fate, we investigated how DNA damage repair and the p53 signal affect the cell fate outcome [41]. In a low ROS environment, few DSBs occur and the number of p53 molecules fluctuates around a steady-state. In rare cases, when a few DSBs are detected by ATM there is a rapid increase in p53 followed by a fall back to the steady-state. As this single pulse of p53 is not enough to induce much p21 transcription, and as a second pulse does not occur because the DSBs have been repaired by then, the downstream early-senescence signalling is not activated. When irradiation is used, many more DSBs occur and the DNA repair machinery is not able to repair all the damage as quickly. If DSBs are present, many sequential pulses of p53 ensue, causing enough p21 to accumulate in the system to induce early senescence signalling. The accumulation of p21 then causes the production of greater levels of ROS via the activation of p38 and GADD45 which decreases DNA repair further and causes more DSBs to form, further increasing p21 and finally pushing the cells into senescence. As our model (calibrated to MCF7 cells, which have been shown to be more resistant to senescence[48]) shows, exposure to 20 Gy irradiation does not lead to complete senescence, with a few cells managing to escape due to the inherent stochasticity in the system. The ability of some cells to evade senescence may be an important step in the initiation of carcinogenesis, since these cells are highly likely to have incorrectly repaired DNA, a feature that often leads to cancer [44,45]. However, further mutations are probably necessary, as we have never witnessed any cancerous outgrowth after 20 Gy irradiation of human fibroblasts.

Although p53 dynamics predicted by our model is very similar to the dynamics measured by Lahav et al [41], there are a few important discrepancies. One is that our model predicts an almost 4-fold higher initial pulse of p53 after irradiation than the following pulses (while there is almost no difference between the pulses in [41]). The other is that the delay in subsequent pulses in our model has more variability than the measured one. Interestingly, when we increased the steady-state abundance of p53 in our simulations to values closer to the measured abundance in MCF7 cells (used in [41]), the difference between the amplitude of the first p53 pulse to other pulses dropped below 2-fold and the time delay between consecutive pulses became more uniform (S5 Fig; 495 ± 130 (low p53) and 475 ± 95 (high p53)). These values are also closer to those measured in another study of p53 oscillations [49]. It is worth noting that to achieve a new higher p53 steady-state, but at the same time keep the senescence outcomes at the same level, as in the original integrated model, we had to change the rates of production and degradation of p53 molecules, thus somewhat changing the dynamics of the original model. Nevertheless, we believe our results indicate that the model is very sensitive to the number of molecules used and that the low number of p53 used in our model might not be adequate for simulating p53 dynamics in cells with higher p53 abundances.

Because sustained p53 signalling was shown to be more efficient in causing senescence than p53 pulsing, we investigated whether repetitive induction of DNA damage would induce senescence differently than single damage events, as has been recently suggested [13]. The model predicted that fractionated low-dose irradiation with 1 Gy would cause a significant number of cells to undergo senescence, although almost no cells undergo senescence when only a single 1 Gy pulse is applied, and that each further irradiation event (up to about 5) would send more cells towards senescence. This is consistent with a study that observed accumulation of DSBs after fractionated therapy [50]. When we sought to confirm this experimentally, the results matched the model’s predictions in broad terms: fractionated irradiation lead to a significant amount of senescence and more pulses (but the same cumulative dose) lead to more senescence; however, the difference between 5x1 and 1x5 Gy pulses was smaller than predicted by modelling and not statistically significant. We currently do not have an explanation for this, but it could be due to different DNA sites, e.g. telomeres, having different repair dynamics [47]. Although others before us have found senescent cells after fractionated irradiation, the irradiation events were further apart (e.g. 5 doses over 2 days), of higher dose (2 Gy or more) and generally achieved much lower senescence (below 5%) [51,52]. Our study suggests that the temporal pattern of irradiation events could play a very important role in radiation therapy and that an integrative modelling approach might therefore be useful for its optimization. In general, the system is controlled by the dynamics of just two components, the damage foci and p53. Most damage foci are permanently resolved within 30 minutes of occurring in MRC5. However the response of p53 is much slower, a pulse triggered by damage may last over 300 minutes. The system, therefore has a rapid trigger for DDR buts its effects endure for ten times longer than the duration of a typical break. As a result, a single burst of damage only triggers a single pulse of p53 unless the damage is not resolved—which is the case for the overwhelming damage of a 20Gy pulse of radiation. However by introducing more bursts of damage (even relatively small ones) the decline in p53 levels is prevented and levels remain elevated driving the production of a more ROS to make more breaks and thereby inducing senescence. During the writing of this paper we have become aware of a new study that looked at the effects of Parp-1 inhibition on radiation sensitivity in cancer cells [53]. The study found that the same cumulative dose of irradiation spread over more time induces more cellular senescence than a quick high dose, and that Parp-1 inhibition can make cells more sensitive to irradiation induced senescence. This partly confirms our modelling predictions and suggests that our modelling approach could also have value in investigating molecular interventions that affect the DNA damage repair or DNA damage signalling pathways.

In summary, the integration of two previously constructed mathematical models has allowed us to make more complete and powerful predictions than with either of the two original models: the senescence feedback was shown to be a plausible explanation for the decreased DNA damage repair that eluded the NHEJ model, while the dynamic DNA damage input from the NHEJ model enabled the senescent model to predict senescence after fractionated irradiation. The size of the original model forced us to abandon SBML and opt for a rule-based approach, which proved very effective both in the ease of integration and in the speed of simulation [25]. Altogether, the model provides a suitable tool for investigating DNA damage induced senescence and can easily be expanded to add more details of the DNA repair pathways [15,21,54], or adapted for modelling other cell fates or cancer therapies that intervene at the level of DNA damage repair or signalling.

## Methods

### Model integration and detailed description

The conversion of the two original SBML models to a single BioNetGen rule-based model was performed by first translating all distinct molecular species within the SBML models into molecules with components (e.g. binding and phosphorylation sites) and states in the BioNetGen language. The following list (see also Fig 1) provides a summary of the molecules, their components and their states in the BioNetGen model (a more detailed explanation can be found in the Supplementary model files):

Source_of_ROS(); ROS(); DNA(id~1~2~3…~50,site~ok~sdsb~cdsb,h2ax~u~p~foci); Ku(dna,cs,cys~red~ox); DNAPKcs(ku,liIV, psite~u~p); LiIV(cs); PARP(dna,liIII); LiIII(PARP); ATM(state~0~1,h2ax); P(); p53_mRNA(); p21_mRNA(); MDM2_mRNA(); GADD45(); p53(psite~u~p); MDM2(psite~u~p); p38(psite~u~p).

In BioNetGen each molecule can have different components, which can either bind to components of other molecules or be in different states. For example, Ku(dna,cs,cys~red~ox) has three components: component dna for binding a DNA molecule, component cs for binding DNA-PKcs and component cys that can be either in the reduced or oxidized state.

As the original models partially overlapped, some reactions needed to be reformatted in the integrated model. For example, in the NHEJ model, ATM and the MRN complex (MRE11A-RAD50-NBN) were single molecules located at a specific site of damage to facilitate phosphorylation of H2AX, which eventually form part of the damage focus. However, in the senescence model, a pool of ATM molecules was activated by DNA damage and in turn caused the phosphorylation of p53 and MDM2. To account for both functions of ATM we modelled it with an active and inactive state (state~0~1) and gave it a binding domain (h2ax) so that it could also become part of the DNA damage foci which is built up and around the phosphorylated H2AX. Within the model the damage foci and the repair complexes form independently of one another but the ligation of a dsb does not occur until both the damage foci and one of the repair complexes have been formed around the site of DNA damage.

Each of the reactions from the original SBML models was then re-created in the rule-based format using the molecules defined above. Rate constants were derived from the original models, although some reactions, such as the recruitment and binding of repair proteins, were combined to change from a two-step process to a one-step process for quicker simulation (Ku70/80, DNA-PKcs, XRCC4, LIG4, Parp-1, XRCC3 and LIG3). We also recreated a timed irradiation event used in the senescent model to simulate the treatment of a cell with irradiation [4]. All the reactions of the integrated model and the reaction rates can be found in S1–S3 Tables.

A typical (reaction) rule in BioNetGen is composed of multiple species that interact and change as a result of their interaction, for example the rule DNA(site!?~sdsb,h2ax~u) + ATM(state~1,h2ax)-> DNA(site!?~sdsb,h2ax~p) + ATM(state~1,h2ax) kh2axp1 can be interpreted as a reaction that occurs between a DNA molecule with a simple DSB (~sdsb) and unphosphorylated h2ax and an ATM molecule in the active state (state~1) with the h2ax binding site free. The reaction leads to phosphorylation of h2ax (h2ax~p) at a rate of kh2axp1. While the DNA molecule has one more component—id, it does not feature in this reaction. This is therefore interpreted as irrelevant for the reaction, in other words the reaction will take place regardless of the state of the id component, therefore this single rule actually codes for 50 different reactions (DNA(id~1,…), DNA(id~2,…),…, DNA(id~50,…)). Another useful syntax is site!?, which is interpreted in the following way: only those DNA molecules, which have something bound (!) to the site can participate in the reaction, however what exactly is bound is not important (?). In our case, Ku70/80or Parp-1 can be bound to the DNA and the reaction takes place with the same reaction rate in both cases.

### Modelling early-senescence

In human fibroblasts p21 is the major driving factor behind the transition into senescence due to DNA damage [55–57]. When p21 increases, the pro-senescence pathway becomes active; however senescence can be avoided if the levels are not kept consistently high. To recreate this behaviour in our model we created a molecule called Sen:

Sen (int~1~10~PLUS~MINUS, State~norm~sen)

Sen has a component int made up of integers between 1 and 10 which increase/decrease depending on the level of p21: high p21 causes the int to increase (PLUS function), while in low levels int decreases (MINUS function) in the rule (S2 Table). If the int component reaches 10 the cell has become senescent and a reaction takes place that changes the second component called State from state norm to sen. The presence of the molecule Sen with the component state sen triggers a number of functions in the model that gradually reduce the levels of Ku70/80 and Parp-1 to levels observed in senescent states and reduce the ability of the Ku complex to bind to a DSB [36].

### Simulation

All simulations were carried out using the network-free stochastic simulator NFsim [28] running on a desktop PC. Non-irradiated cells were simulated for 30 hours. Irradiated cells were simulated for 3 hours prior to irradiation, and for 78 hours after irradiation, which matched the corresponding experimental data and also allowed the simulation of the early senescent feedback. The parameters chosen for recalibration were recalibrated using relevant experimental data and the fmincon optimization function in Matlab (Matlab 2010b, MathWorks, Massachusetts, United States) using the default settings (the model was run > 500 times for evaluation). Among the parameters varied in the simulations were the levels of environmental ROS for the non-irradiated cells, the level of the irradiation exposure for the irradiated cells, and finally the number and frequency of irradiation events for the study of senescence after fractionated irradiation.

### Cell culture

Human MRC5 fibroblasts (obtained from ECACC) were cultured in Dulbecco’s modified Eagle’s medium (DMEM; Sigma, Dorset, UK) enriched with 10% heat inactivated foetal bovine serum (FBS; BioSera, Ringmer, UK), 2mM L-Glutamine and 1% penicillin/streptomycin. Fibroblasts were grown on 150 cm^2^ flasks (Corning Incorporated, Corning, NY, USA) in a humified atmosphere of 5% CO_2_, 20% O_2_ and 95% air at 37°C. Cells were split at 90% confluence into identical fresh medium.

### Irradiation

Cells were seeded onto 18 mm Ø No 1.5 glass coverslips 48 hours before irradiation and grown to 80% confluence over 48 hours as described above. Coverslips to be irradiated were subjected to a 1 or 5 Gy irradiation pulse in a XRAD225 Biological Irradiator (Precision X-ray Inc, N Branford, CT, USA). Media for each slide was then replaced and slides left for an hour before a repeat pulse was delivered (where applicable).

### Sen β gal staining

Slides were washed in phosphate buffer solution (PBS) and fixed 96 hours after the start of irradiation protocol in 2% paraformaldehyde in PBS for 5 minutes. Slides were then washed and stored in PBS. Before staining slides were washed in 5 mM MgCl_2_ in PBS (PBS-Mg). Cells were stained for senescence overnight using Sen β gal staining solution (5 mM MgCl_2_, 50 mM K_4_Fe(CN)_6_·3H_2_O, 50 mM K_3_[Fe(CN)_6_], 1mg/ml X-gal (Sigma) in PBS at pH 5.7). The next day slides were washed with PBS-Mg and mounted onto microscope slides using Vectashield Mounting Medium with 4',6-diamidino-2-phenylindole (DAPI) nuclear staining (Vector Laboratories, Peterborough, UK).

### Cell imaging

Cells were imaged with a Leica DFC420 camera (Leica Microsystems UK, Milton Keynes, UK) mounted to a Nikon Eclipse E800 microscope with a Nikon Plan Fluor 40x/0.75 air objective (Nikon UK, Kingston Upon Thames, UK). Areas were selected for counting using only the DAPI stain (excitation at 340–380 nm, emission 425nm) so as not to bias the results. Both DAPI fluorescence and Brightfield images were taken for senescence analysis. The two sets of images were then overlaid in Image J so that the presence of DAPI around the nucleus of a cell could be detected. Presence of the stain within the cell indicated a transition to a senescent state.

### ROS measurements

To better calibrate the levels of ROS production during senescence in the model we measured the rates of the release of hydrogen peroxide (H_2_O_2_) from cultured cells using Amplex Red reagent (Invitrogen, A12222). H_2_O_2_ is the most common source of damage to DNA as it has a long half-life and is mobile including being able to diffuse across cellular membranes.

Amplex Red (10-acetyl-3,7-dihydroxyphenoxazine) reacts with H_2_O_2_ in a 1:1 stoichiometry to produce the red-fluorescent oxidation product, resorufin, in the presence of horseradish peroxidase. The cells were trypsinised and resuspended in culture media, and the time courses of resorufin appearance were followed fluorometrically at 37°C at an excitation 544 nm and an emission 590 nm in a black bottom 96 well plate using a FLUOstar Omega (BMG Labtech). Each well consisted of 150,000cells, 50μM Amplex Red and 2U/ml horseradish peroxidase. Known amounts of H_2_O_2_ were added to blank wells to construct the H_2_O_2_ standard curve in order to convert the fluorescence arbitrary units to moles of H_2_O_2_.

## Supporting Information

S1 Text List of all molecules and their components used in the rule-based model. (DOCX)

S2 Text Definition of a pulse. (DOCX)

S1 Fig DNA damage foci longevity. Histogram of the recorded foci longevities for live cell and simulation for non-irradiated cells (A) and irradiated cells (B) corresponding to data presented in Fig 2.
(TIF)

S2 Fig Simulation averages across population of irradiated cells. The average time course for p21, p53, DNA damage foci and ROS molecules for cells after exposure to different level of irradiation.
(TIF)

S3 Fig Single cell simulations after fractionated irradiation. Time courses for ROS, DNA damage foci, p53 and p21 for a representative 5 x 1 Gy simulation of the integrated model.
(TIF)

S4 Fig Simulation averages of population of cells after different temporal patterns of irradiation. The average time course for p21, p53, DNA damage foci and ROS after irradiation with a single 5 Gy irradiation pulse, 5 x 1 Gy pulses with 1 hour between the pulses and 5 x 1 Gy pulses with 2 hours between the pulses.
(TIF)

S5 Fig Single cell simulations of irradiated cell with high p53 abundance. The time course for number of molecules of p53 for two representative simulations of the integrated model with high p53 abundance is shown.
(TIFF)

S1 Table Rules in the model. For brevity only rules for species DNA1 are shown, however within the model there are corresponding rules for 50 DNA molecules (DNA1 through to DNA50).
(DOCX)

S2 Table Functions in the model. Functions allow for different rate constants to be utilised by a rule if certain conditions are met. A # within the units of the rate constant stands for number (of particles/individuals), so a rate constant with a unit of #^-1^min^-1^ would be per number per minute.
(DOCX)

S3 Table Rate constants. Most rate constants within the model are from the original SBML models that the rule based model was developed from. The merged rates from Dolan et al 2013 are a composite of rate constants from a number of reactions that were merged to help keep the reaction based model from being needlessly large. The new rate constants were created due to the introduction of new reactions where the two original models were merged together, or were experimentally determined. A # within the units of the rate constant stands for number (of particles/individuals), so a rate constant with a unit of #^-1^min^-1^ would be per number per minute.
(DOCX)

S1 Model Mathematical model of DNA damage and early senescence signalling in BioNetGen format. (BNGL)
