# Supplementary material for: Integrated Stochastic Model of DNA Damage Repair by Non-homologous End Joining and p53/p21- Mediated Early Senescence Signalling
Source: PLoS Comput Biol. 2015 May 28;11(5):e1004246. doi: 10.1371/journal.pcbi.1004246 (PMC4447392; doi:10.1371/journal.pcbi.1004246)
Supplement: S1 Text — (DOCX) [file pcbi.1004246.s001.docx]

**S1 Text. Molecules and their components.**

In the integrated rule-based model, the Source of ROS and ROS itself have no components as they are involved in simple reactions and are not modified nor do they bind to anything. The DNA species were assigned a numbered ID so that each DSB could undergo reactions and be monitored individually. The DSB was defined by creating a DNA component called ‘site’ which could be in an undamaged state (ok), have a simple DSB (sdsb) or a complex DSB (cdsb). Whereas the original NHEJ model could only accommodate twenty theoretical break sites, in the rule based model we were able to increase this to fifty sites. As the histone H2AX is part of a DNA chain we made it a component of the DNA species called h2ax that could be in one of three states, unphosphorylated (u), phosphorylated (p) and within a damage foci (foci) rather than model it as a distinct species. The Ku 70/80 heterodimer required a component to represent its binding to the DSB (dna); another to allow for the binding of DNA-PKcs to form the DNA-PK complex (cs); and finally a component to represent whether cysteine 289 (cys) was in a reduced (red) or an oxidised (ox) state. The DNA-PKcs needed components to represent its binding to Ku70/80 (ku), LiIV (liIV) and a third to indicate whether it had undergone auto-phosphorylation (psite~u~p). LiIV was given a single component to represent binding to DNA-PKcs. PARP-1 was assigned a component to facilitate its binding to the DSB (dna) and another to allow the binding of LiIII (liIII) and the LiIII molecule itself was given a single component so that it could be bound to PARP-1 (PARP). All mRNA molecules and GADD45 were modelled without any components as they do not undergo alterations or bindings in the original model. p53, MDM2 and p38 were given a component with an unphosphorylated and phosphorylated state (psite~u~p). Since p21 was produced in 2 steps in the senescence model to simulate its translation dynamics, we gave it a component called step with 3 states (1, 2 and 3) where state 3 would represent the final product of the p21 synthesis.
