# Supplementary material for: Integrated Stochastic Model of DNA Damage Repair by Non-homologous End Joining and p53/p21- Mediated Early Senescence Signalling
Source: PLoS Comput Biol. 2015 May 28;11(5):e1004246. doi: 10.1371/journal.pcbi.1004246 (PMC4447392; doi:10.1371/journal.pcbi.1004246)
Supplement: S2 Text — (DOCX) [file pcbi.1004246.s002.docx]

**S2 Text. Definition of pulse.**

In their 2004 study Lahav et al have analyzed single cell p53 signalling by measuring the properties of p53 pulses, however in their publication we could not find an explicit definition of a pulse or its width nor have they made it clear whether the pulse measurements were done by hand or by algorithm [40]. In this study, we defined a p53 pulse as any signal with amplitude larger than mean + 2.5*SD of the simulated p53 signal without irradiation (i.e., every time the number of p53 molecules is over 285). The factor 2.5 was determined by matching the number of experimentally determined pulses after irradiation with 0.3 Gy with our simulations (not used in Figure3a). We defined the pulse width as the time between the points when p53 levels started to increase for the last time before reaching the pulse peak to the time it stopped decreasing after the peak. We ignored any increases or decreases that lasted for less than 15 minutes.
