# Supplementary figures and images for: Integrated Stochastic Model of DNA Damage Repair by Non-homologous End Joining and p53/p21- Mediated Early Senescence Signalling
Source: PLoS Comput Biol. 2015 May 28;11(5):e1004246. doi: 10.1371/journal.pcbi.1004246 (PMC4447392; doi:10.1371/journal.pcbi.1004246)

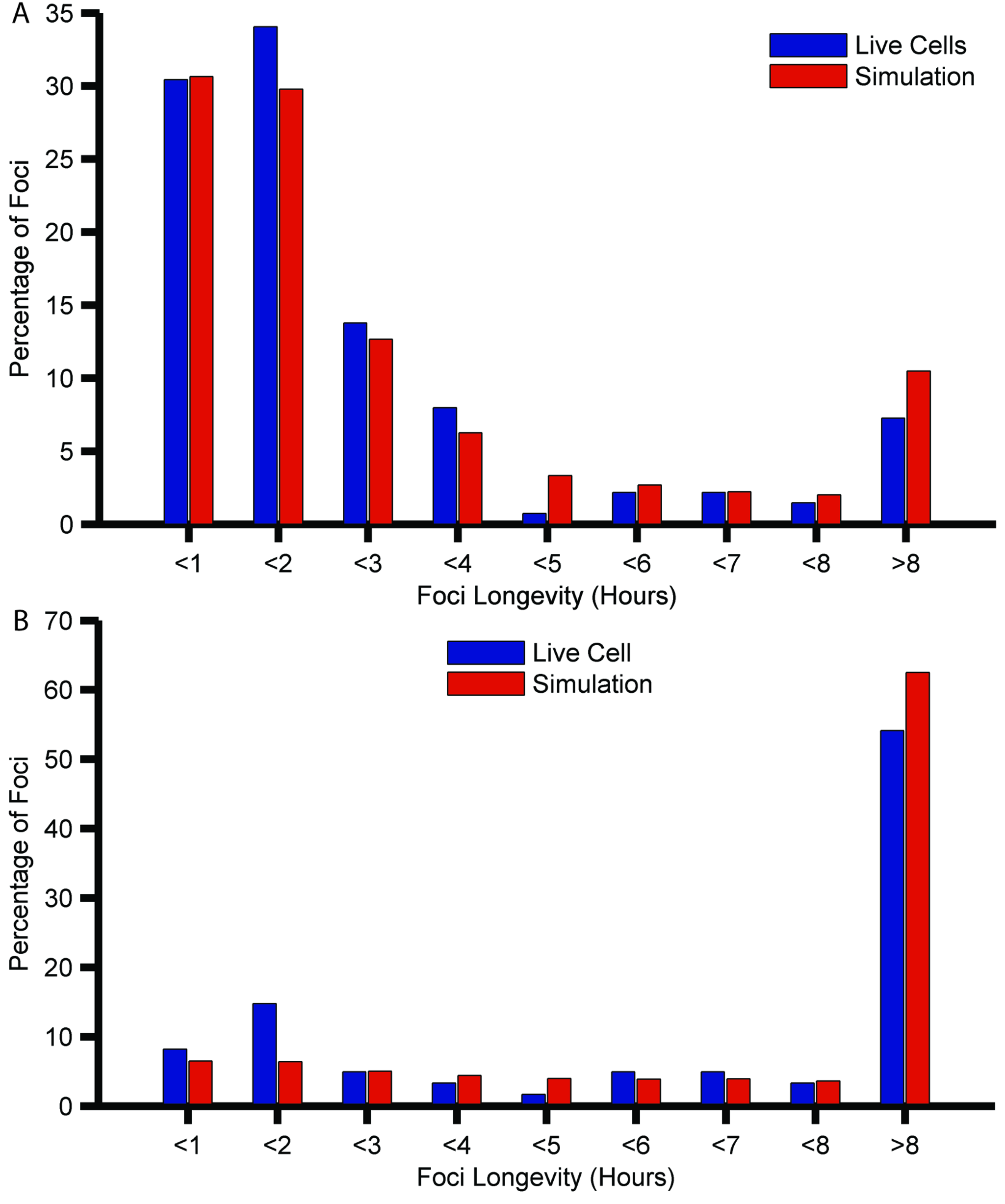

Supplement: S1 Fig — Histogram of the recorded foci longevities for live cell and simulation for non-irradiated cells (A) and irradiated cells (B) corresponding to data presented in Fig 2. (TIF) [file pcbi.1004246.s003.tif]

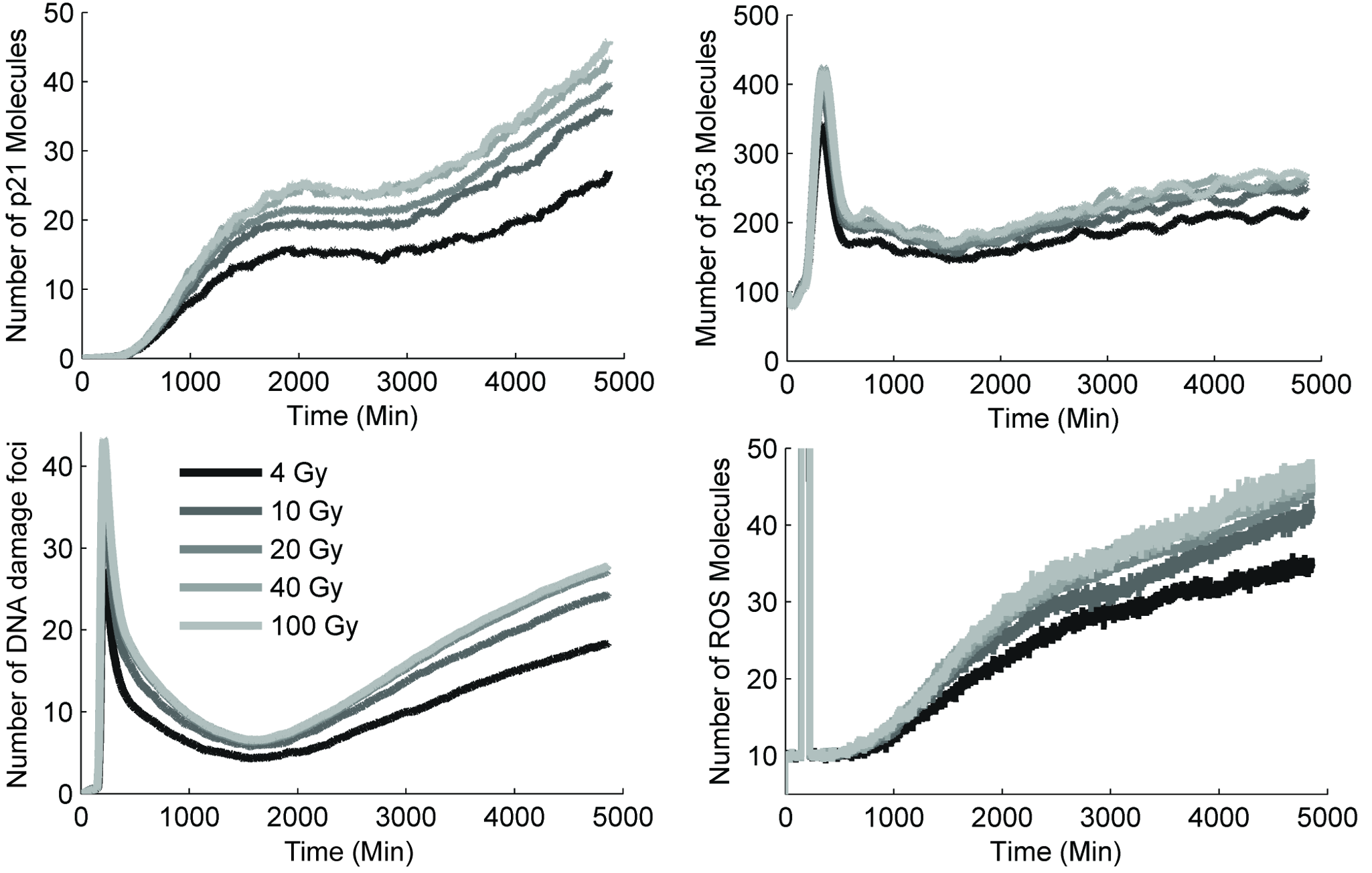

Supplement: S2 Fig — The average time course for p21, p53, DNA damage foci and ROS molecules for cells after exposure to different level of irradiation. (TIF) [file pcbi.1004246.s004.tif]

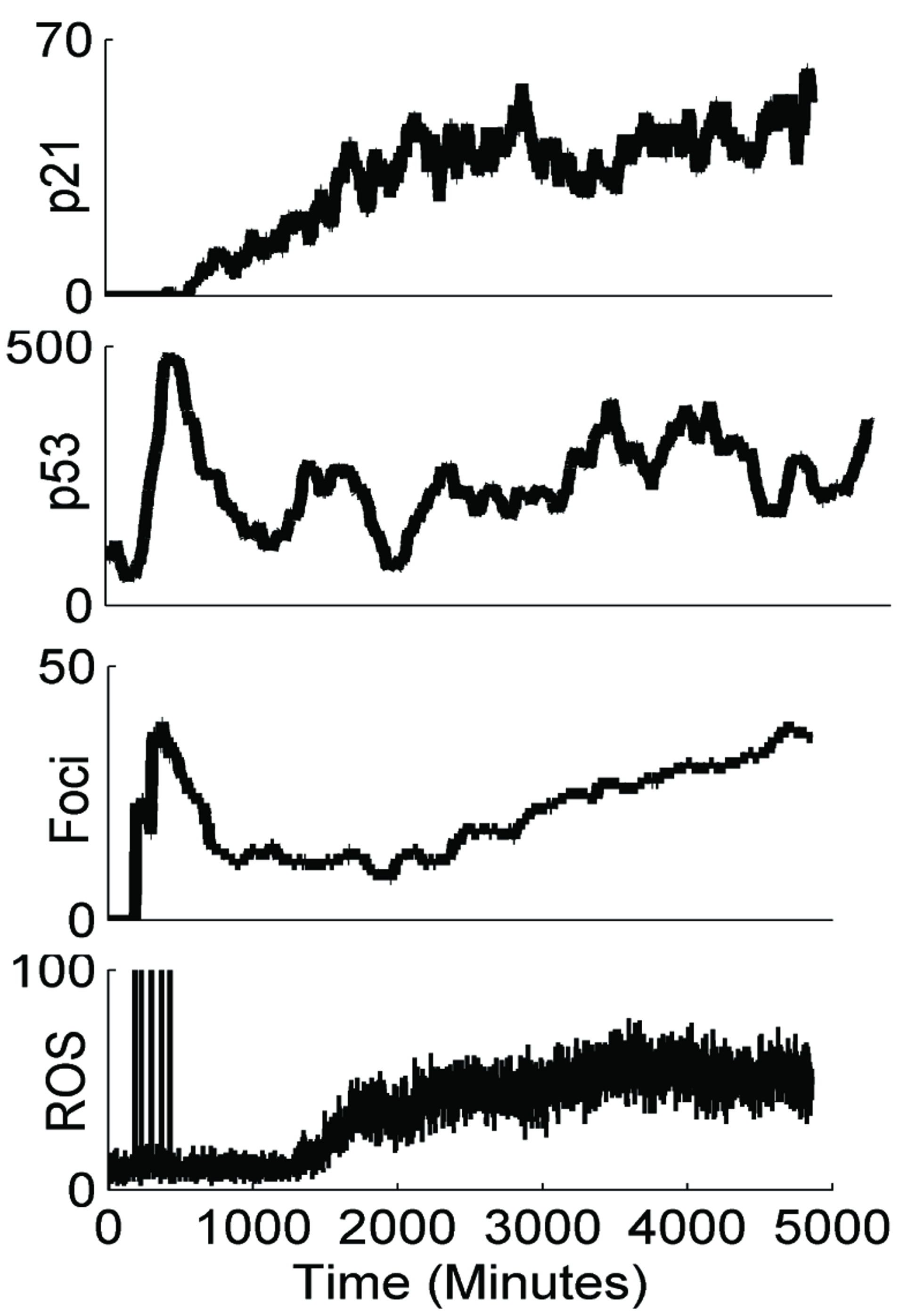

Supplement: S3 Fig — Time courses for ROS, DNA damage foci, p53 and p21 for a representative 5 x 1 Gy simulation of the integrated model. (TIF) [file pcbi.1004246.s005.tif]

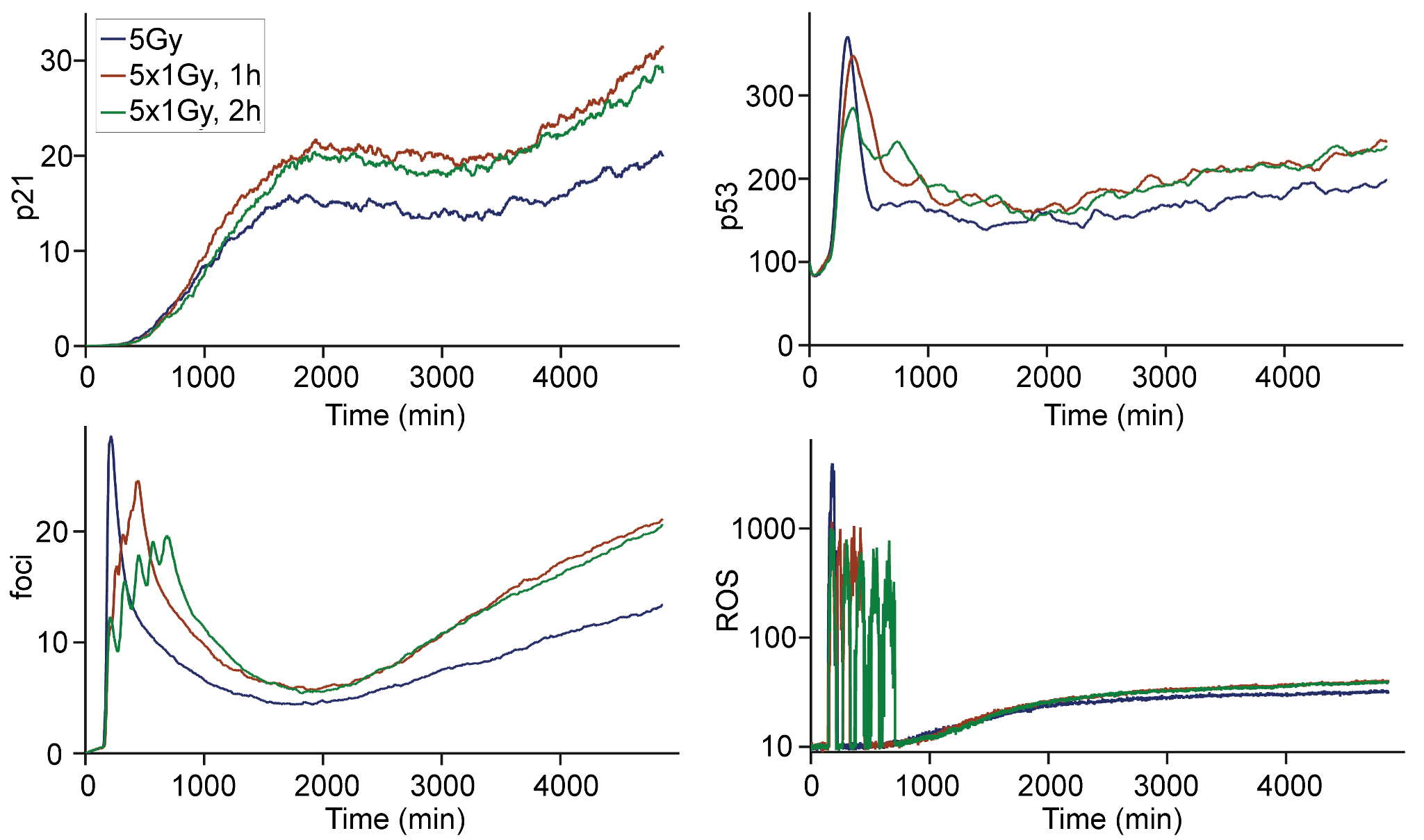

Supplement: S4 Fig — The average time course for p21, p53, DNA damage foci and ROS after irradiation with a single 5 Gy irradiation pulse, 5 x 1 Gy pulses with 1 hour between the pulses and 5 x 1 Gy pulses with 2 hours between the pulses. (TIF) [file pcbi.1004246.s006.tif]

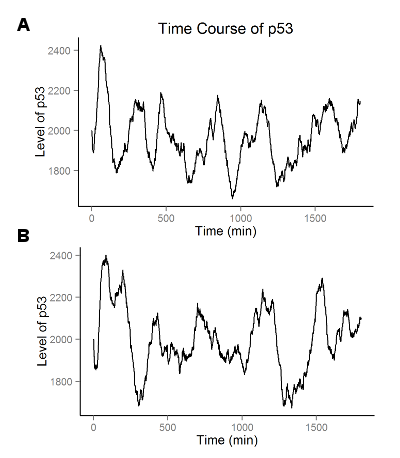

Supplement: S5 Fig — The time course for number of molecules of p53 for two representative simulations of the integrated model with high p53 abundance is shown. (TIFF) [file pcbi.1004246.s007.tiff]
