# Supplementary material for: Integrated Stochastic Model of DNA Damage Repair by Non-homologous End Joining and p53/p21- Mediated Early Senescence Signalling
Source: PLoS Comput Biol. 2015 May 28;11(5):e1004246. doi: 10.1371/journal.pcbi.1004246 (PMC4447392; doi:10.1371/journal.pcbi.1004246)
Supplement: S1 Table — For brevity only rules for species DNA1 are shown, however within the model there are corresponding rules for 50 DNA molecules (DNA1 through to DNA50). (DOCX) [file pcbi.1004246.s008.docx]

Table S1

| Reaction | BioNetGen Rules |
| --- | --- |
| p53 mRNA Synthesis | P() -> p53_mRNA() + P() kp53mRNAsyn |
| p53 mRNA Degradation | p53_mRNA() -> Sink() kp53mRNAdeg |
| p53 Synthesis | p53_mRNA() -> p53_mRNA() + p53(Site1~u) kp53syn |
| p53 Degradation | p53(Site1~u) -> Sink() kp53deg  p53(Site1~p) -> Sink() kp53deg  p53(Site1!1~u).MDM2(Site1!1~u) -> MDM2(Site1~u) kp53degMDM2dep DeleteMolecules |
| p53 Phosphorylation | p53(Site1~u) + ATM(state~1,h2ax!?) -> p53(Site1~p) + ATM(state~1,h2ax!?) kp53phos |
| p53 Dephosphorylation | p53(Site1~p) -> p53(Site1~u) kp53dphos |
| MDM2 mRNA Synthesis | p53(Site1~u) -> MDM2_mRNA() + p53(Site1~u) kMDM2mRNAsyn  p53(Site1~p) -> MDM2_mRNA() + p53(Site1~p) kMDM2mRNAsyn |
| MDM2 mRNA Degradation | MDM2_mRNA() -> Sink() kMDM2mRNAdeg |
| MDM2 Synthesis | MDM2_mRNA() -> MDM2_mRNA() + MDM2(Site1~u) kMDM2syn |
| MDM2 Degradation | MDM2(Site1~u) -> Sink() kMDM2deg  MDM2(Site1~p) -> Sink() kMDM2pdeg |
| MDM2 Phosphorylation | MDM2(Site1~u) + ATM(state~1,h2ax!?) -> MDM2(Site1~p) + ATM(state~1,h2ax!?) kMDM2phos |
| MDM2 Dehosphorylation | MDM2(Site1~p) -> MDM2(Site1~u) kMDM2dphos |
| p53-MDM2 Binding | p53(Site1~u) + MDM2(Site1~u) -> p53(Site1!1~u).MDM2(Site1!1~u) kp53MDM2bind |
| p53-MDM2 Dissociation | p53(Site1!1~u).MDM2(Site1!1~u) -> p53(Site1~u) + MDM2(Site1~u) kp53MDM2dis |
| p21 mRNA Synthesis | p53(Site1~u) -> p21_mRNA() + p53(Site1~u) kp21mRNAsyn  p53(Site1~p) -> p21_mRNA() + p53(Site1~p) kp21mRNAsynp |
| p21 mRNA Degradation | p21_mRNA() -> Sink() kp21mRNAdeg |
| p21 Synthesis | p21_mRNA() -> p21(step~1) + p21_mRNA() kp21synstep1  p21(step~1) -> p21(step~2) kp21synstep2  p21(step~2) -> p21(step~3) kp21synstep3 |
| p21 Degradation | p21(step~3) -> Sink() kp21deg |
| GADD45 Activation | p21(step~3) -> p21(step~3) + GADD45() kGADD45act |
| GADD45 Degradation | GADD45() -> Sink() kGADD45deg |
| p38 Phosphorylation | p38(Site1~u) + GADD45() -> p38(Site1~p) + GADD45() kp38phos |
| p38 Dephosphorylation | p38(Site1~p) -> p38(Site1~u) kp38dphos |
| p38 ROS Generation | p38(Site1~p) -> ROS() + p38(Site1~p) kROSgen |
| Ku-DSB Binding | DNA(site~sdsb) + Ku(dna,cs) -> DNA(site!1~sdsb).Ku(dna!1,cs) kku1()  DNA(site~cdsb) + Ku(dna,cs) -> DNA(site!1~cdsb).Ku(dna!1,cs) kku2() |
| Ku-DSB Dissociation | DNA(site!1~sdsb).Ku(dna!1,cs,cys~red) -> DNA(site~sdsb) + Ku(dna,cs,cys~red) kdku1  DNA(site!1~cdsb).Ku(dna!1,cs,cys~red) -> DNA(site~cdsb) + Ku(dna,cs,cys~red) kdku2 |
| DNA-PK Complex Formation | DNA(site!1~sdsb).Ku(dna!1,cs) + DNAPKcs(ku,liIV,psite~u) -> DNA(site!1~sdsb).Ku(dna!1,cs!2).DNAPKcs(ku!2,liIV,psite~u) kdnapk1  DNA(site!1~cdsb).Ku(dna!1,cs) + DNAPKcs(ku,liIV,psite~u) -> DNA(site!1~cdsb).Ku(dna!1,cs!2).DNAPKcs(ku!2,liIV,psite~u) kdnapk2 |
| DNA-PK Complex Dissociation | DNA(site!1~sdsb).Ku(dna!1,cs!2).DNAPKcs(ku!2,liIV,psite~?) -> DNA(site~sdsb) + Ku(dna,cs) + DNAPKcs(ku,liIV,psite~u) kddnapk1  DNA(site!1~cdsb).Ku(dna!1,cs!2).DNAPKcs(ku!2,liIV,psite~?) -> DNA(site~cdsb) + Ku(dna,cs) + DNAPKcs(ku,liIV,psite~u) kddnapk2 |
| DNAPKcs Phosphorylation | DNA(site!1~sdsb).Ku(dna!1,cs!2).DNAPKcs(ku!2,liIV,psite~u) -> DNA(site!1~sdsb).Ku(dna!1,cs!2).DNAPKcs(ku!2,liIV,psite~p) kdnapkphos1  DNA(site!1~cdsb).Ku(dna!1,cs!2).DNAPKcs(ku!2,liIV,psite~u) -> DNA(site!1~cdsb).Ku(dna!1,cs!2).DNAPKcs(ku!2,liIV,psite~p) kdnapkphos2 |
| Ligase IV Complex Formation | DNA(site!1~sdsb).Ku(dna!1,cs!2).DNAPKcs(ku!2,liIV,psite~p) + LiIV(cs) ->  DNA(site!1~sdsb).Ku(dna!1,cs!2).DNAPKcs(ku!2,liIV!3,psite~p).LiIV(cs!3) kliIV1  DNA(site!1~cdsb).Ku(dna!1,cs!2).DNAPKcs(ku!2,liIV,psite~p) + LiIV(cs) ->  DNA(site!1~cdsb).Ku(dna!1,cs!2).DNAPKcs(ku!2,liIV!3,psite~p).LiIV(cs!3) kliIV2 |
| LiIV Dissociation | DNA(site!1~sdsb).Ku(dna!1,cs!2).DNAPKcs(ku!2,liIV!3,psite~p).LiIV(cs!3) ->  DNA(site!1~sdsb).Ku(dna!1,cs!2).DNAPKcs(ku!2,liIV,psite~p) + LiIV(cs) kdliIV1  DNA(site!1~cdsb).Ku(dna!1,cs!2).DNAPKcs(ku!2,liIV!3,psite~p).LiIV(cs!3) ->  DNA(site!1~cdsb).Ku(dna!1,cs!2).DNAPKcs(ku!2,liIV,psite~p) + LiIV(cs) kdliIV2 |
| Repair | DNA(site!1~sdsb,h2ax!4~foc).Ku(dna!1,cs!2).DNAPKcs(ku!2,liIV!3,psite~p).LiIV(cs!3).ATM(h2ax!4) ->  DNA(site~ok,h2ax!1~foc).ATM(h2ax!1) + Ku(dna,cs) + DNAPKcs(ku,liIV,psite~u) + LiIV(cs) kfixIV1  DNA(site!1~cdsb,h2ax!4~foc).Ku(dna!1,cs!2).DNAPKcs(ku!2,liIV!3,psite~p).LiIV(cs!3).ATM(h2ax!4) ->  DNA(site~ok,h2ax!1~foc).ATM(h2ax!1) + Ku(dna,cs) + DNAPKcs(ku,liIV,psite~u) + LiIV(cs) kfixIV2 |
| PARP Binding | DNA(site~sdsb) + PARP(dna,liIII) -> DNA(site!1~sdsb).PARP(dna!1,liIII) kPARP1  DNA(site~cdsb) + PARP(dna,liIII) -> DNA(site!1~cdsb).PARP(dna!1,liIII) kPARP2 |
| PARP Dissociation | DNA(site!1~sdsb).PARP(dna!1,liIII) -> DNA(site~sdsb) + PARP(dna,liIII) kdPARP1  DNA(site!1~cdsb).PARP(dna!1,liIII) -> DNA(site~cdsb) + PARP(dna,liIII) kdPARP2 |
| Ligase III Complex Formation | DNA(site!1~sdsb).PARP(dna!1,liIII) + LiIII(PARP) -> DNA1(site!1~sdsb).PARP(dna!1,liIII!2).LiIII(PARP!2) kliIII1  DNA1(site!1~cdsb).PARP(dna!1,liIII) + LiIII(PARP) -> DNA1(site!1~cdsb).PARP(dna!1,liIII!2).LiIII(PARP!2) kliIII2 |
| LiIII Dissociation | DNA1(site!1~sdsb).PARP(dna!1,liIII!2).LiIII(PARP!2) -> DNA1(site!1~sdsb).PARP(dna!1,liIII) + LiIII(PARP) kdliIII1  DNA1(site!1~cdsb).PARP(dna!1,liIII!2).LiIII(PARP!2) -> DNA1(site!1~cdsb).PARP(dna!1,liIII) + LiIII(PARP) kdliIII2 |
| Repair (LiIII) | DNA1(site!1~sdsb,h2ax!3~foc).PARP(dna!1,liIII!2).LiIII(PARP!2).ATM(h2ax!3) ->  DNA1(site~ok,h2ax!1~foc).ATM(h2ax!1) + PARP(dna,liIII) + LiIII(PARP) kfixIII1  DNA1(site!1~cdsb,h2ax!3~foc).PARP(dna!1,liIII!2).LiIII(PARP!2).ATM(h2ax!3) ->  DNA1(site~ok,h2ax!1~foc).ATM(h2ax!1) + PARP(dna,liIII) + LiIII(PARP) kfixIII2  DNA1(site!1~sdsb,h2ax!3~foc).PARP(dna!1,liIII!2).LiIII(PARP!2).ATM(h2ax!3) ->  DNA1(site~ok,h2ax!1~foc).ATM(h2ax!1) + PARP(dna,liIII) + LiIII(PARP) kfixIII3  DNA1(site!1~cdsb,h2ax!3~foc).PARP(dna!1,liIII!2).LiIII(PARP!2).ATM(h2ax!3) ->  DNA1(site~ok,h2ax!1~foc).ATM(h2ax!1) + PARP(dna,liIII) + LiIII(PARP) kfixIII4 |
| ATM Activation | DNA1(site!?~sdsb) + ATM(state~0,h2ax) -> DNA1(site!?~sdsb) + ATM(state~1,h2ax) kATMact  DNA1(site!?~cdsb) + ATM(state~0,h2ax) -> DNA1(site!?~cdsb) + ATM(state~1,h2ax) kATMact |
| ATM Inactivation | ATM(state~1,h2ax) -> ATM(state~0,h2ax) kATMinact |
| Histone Phosphorylation | DNA1(site!?~sdsb,h2ax~u) + ATM(state~1,h2ax) -> DNA1(site!?~sdsb,h2ax~p) + ATM(state~1,h2ax) kh2axp1  DNA1(site!?~cdsb,h2ax~u) + ATM(state~1,h2ax) -> DNA1(site!?~cdsb,h2ax~p) + ATM(state~1,h2ax) kh2axp1  DNA1(site!1~sdsb,h2ax~u).Ku(dna!1,cs!2).DNAPKcs(ku!2,liIV,psite~p) ->  DNA1(site!1~sdsb,h2ax~p).Ku(dna!1,cs!2).DNAPKcs(ku!2,liIV,psite~p) kh2axp2  DNA1(site!1~cdsb,h2ax~u).Ku(dna!1,cs!2).DNAPKcs(ku!2,liIV,psite~p) ->  DNA1(site!1~cdsb,h2ax~p).Ku(dna!1,cs!2).DNAPKcs(ku!2,liIV,psite~p) kh2axp2 |
| Histone Dephosphorylation | DNA1(h2ax~p) -> DNA1(h2ax~u) kh2axu |
| Foci Formation | DNA1(h2ax~p) -> DNA1(h2ax~foc) kh2axfoc |
| Foci Dephosphorylation | DNA1(h2ax~foc) -> DNA1(h2ax~u) kfocback |
| Complete Foci Formation | DNA1(h2ax~foc) + ATM(state~1,h2ax) -> DNA1(h2ax!1~foc).ATM(state~1,h2ax!1) kh2axfull |
| Foci Resolution | DNA1(h2ax!1~foc).ATM(state~1,h2ax!1) -> DNA1(h2ax~u) + ATM(state~0,h2ax) kfocfin |
| ROS production | I() -> I() + ROS() kROS  ROS() -> Sink() kdROS  IR() -> IR() + ROS() kIR |
| DNA Damage | DNA1(site~ok) + ROS() -> DNA1(site~sdsb) kdam1  DNA1(site~ok) + ROS() -> DNA1(site~cdsb) kdam2 |
| Ku Redox | Ku(dna,cs,cys~red) + ROS() -> Ku(dna,cs,cys~ox) + ROS() kox  Ku(dna,cs,cys~ox) -> Ku(dna,cs,cys~red) kred |
| Ku-DSB Dissociation (Oxidised) | DNA1(site!1~sdsb).Ku(dna!1,cs,cys~ox) -> DNA1(site~sdsb) + Ku(dna,cs,cys~ox) kdku3  DNA1(site!1~cdsb).Ku(dna!1,cs,cys~ox) -> DNA1(site~cdsb) + Ku(dna,cs,cys~ox) kdku4 |
| Senescent Shift Rules | Sen(int~?,State~normal) -> Sen(int~PLUS,State~normal) kplus()  Sen(int~?,State~normal) -> Sen(int~MINUS,State~normal) kminus()  Sen(int~10,State~normal) -> Sen(int~10,State~sen) ksen #Make ksen very big  Ku(dna,cs,cys~?) -> Sink() kKuDown() * kKustop()  PARP(dna,liIII) -> Sink() kParpDown() * kParpstop() |
