# Supplementary material for: Integrated Stochastic Model of DNA Damage Repair by Non-homologous End Joining and p53/p21- Mediated Early Senescence Signalling
Source: PLoS Comput Biol. 2015 May 28;11(5):e1004246. doi: 10.1371/journal.pcbi.1004246 (PMC4447392; doi:10.1371/journal.pcbi.1004246)
Supplement: S2 Table — Functions allow for different rate constants to be utilised by a rule if certain conditions are met. A # within the units of the rate constant stands for number (of particles/individuals), so a rate constant with a unit of #-1min-1 would be per number per minute. (DOCX) [file pcbi.1004246.s009.docx]

| Function | Units of Rate Constants | Function Purpose |
| --- | --- | --- |
| kku1() = if(Senescent_Counter>0,0.000000034,0.00034) | #^-1^min^-1^ | Reduces the association rate of Ku to a simple DSB during Senescence |
| kku2() = if(Senescent_Counter>0,0.000000021,0.00021) | #^-1^min^-1^ | Reduces the association rate of Ku to a complex DSB during Senescence |
| kplus() = if(p21>15,0.04,0) | min^-1^ | Promotes the switch to senescence based on abundance of p21 |
| kminus() = if(p21<=15&&Sen_Min<1,0.5,0) | min^-1^ | Impedes the switch to senescence based on abundance of p21 |
| kKuDown() = if(Senescent_Counter==1,0.01,0) | min^-1^ | Decreases Ku levels depending on senescent state |
| kKustop() = if(Ku<=250,0,1) | min^-1^ | Stops decrease in Ku levels below 250 molecules |
| kParpDown() = if(Senescent_Counter==1,0.01,0) | min^-1^ | Decreases PARP levels depending on senescent state |
| kParpstop() = if(PARP<=5,0,1) | min^-1^ | Stops decrease in PARP levels below 5 molecules |
| Irrad()= if(Time<180&&Time>174,2000,0) | min^-1^ | Triggers Irradiation event |
| IRoff()= if(Time>=180,1000,0) | min^-1^ | Stops Irradiation event |
