# Supplementary material for: Integrated Stochastic Model of DNA Damage Repair by Non-homologous End Joining and p53/p21- Mediated Early Senescence Signalling
Source: PLoS Comput Biol. 2015 May 28;11(5):e1004246. doi: 10.1371/journal.pcbi.1004246 (PMC4447392; doi:10.1371/journal.pcbi.1004246)
Supplement: S3 Table — Most rate constants within the model are from the original SBML models that the rule based model was developed from. The merged rates from Dolan et al 2013 are a composite of rate constants from a number of reactions that were merged to help keep the reaction based model from being needlessly large. The new rate constants were created due to the introduction of new reactions where the two original models were merged together, or were experimentally determined. A # within the units of the rate constant stands for number (of particles/individuals), so a rate constant with a unit of #-1min-1 would be per number per minute. (DOCX) [file pcbi.1004246.s010.docx]

Table S3.

| Rate Constant ID | Rate | Description | Source |
| --- | --- | --- | --- |
| kp53mRNAsyn | 0.06 min^-1^ | Rate of p53 mRNA Synthesis | Passos *et al* 2010 |
| kp53mRNAdeg | 0.006 min^-1^ | Rate of p53 mRNA Degradation | Passos *et al* 2010 |
| kp53syn | 0.36 min^-1^ | Rate of p53 Synthesis | Passos *et al* 2010 |
| kp53deg | 4.95E-05 min^-1^ | Rate of p53 Degradation | Passos *et al* 2010 |
| kp53degMDM2dep | 0.0495 min^-1^ | Rate of p53 Degredation-MDM2 Dependant | Passos *et al* 2010 |
| kp53phos | 0.36 #^-1^min^-1^ | Rate of p53 Phosphorylation | Passos *et al* 2010 |
| kp53dphos | 30 min^-1^ | Rate of p53 Dephosphorylation | Passos *et al* 2010 |
| kMDM2mRNAsyn | 0.006 min^-1^ | Rate of MDM2 mRNA Synthesis | Passos *et al* 2010 |
| kMDM2mRNAdeg | 0.006 min^-1^ | Rate of MDM2 mRNA Degradation | Passos *et al* 2010 |
| kMDM2syn | 0.0297 min^-1^ | Rate of MDM2 Synthesis | Passos *et al* 2010 |
| kMDM2deg | 0.02598 min^-1^ | Rate of MDM2 Degradation | Passos *et al* 2010 |
| kMDM2pdeg | 0.024 min^-1^ | Rate of Phosphorylated MDM2 Degredation | Passos *et al* 2010 |
| kMDM2phos | 120 #^-1^min^-1^ | Rate of MDM2 Phosphorylation | Passos *et al* 2010 |
| kMDM2dphos | 30 min^-1^ | Rate of p53 Dephosphorylation | Passos *et al* 2010 |
| kp53MDM2bind | 0.0693 #^-1^min^-1^ | Rate of p53 MDM2 Binding | Passos *et al* 2010 |
| kp53MDM2dis | 6.93E-05 min^-1^ | Rate of p53 MDM2 Dissociation | Passos *et al* 2010 |
| kp21mRNAsyn | 3.6E-06 min^-1^ | Rate of p21 mRNA Synthesis (p53) | Passos *et al* 2010 |
| kp21mRNAsynp | 0.00036 min^-1^ | Rate of p21 mRNA Synthesis (Phosphorylated p53) | Passos *et al* 2010 |
| kp21mRNAdeg | 0.00144 min^-1^ | Rate of p21 mRNA Degradation | Passos *et al* 2010 |
| kp21synstep1 | 0.024 min^-1^ | Rate of p21 Synthesis Step 1 | Passos *et al* 2010 |
| kp21synstep2 | 0.0024 min^-1^ | Rate of p21 Synthesis Step 2 | Passos *et al* 2010 |
| kp21synstep3 | 0.0024 min^-1^ | Rate of p21 Synthesis Step 3 | Passos *et al* 2010 |
| kp21deg | 0.0114 min^-1^ | Rate of p21 Degradation | Passos *et al* 2010 |
| kGADD45act | 0.00024 min^-1^ | Rate of GADD45 Activation/Production | Passos *et al* 2010 |
| kGADD45deg | 0.0006 min^-1^ | Rate of GADD45 Degradation | Passos *et al* 2010 |
| kp38phos | 0.48 #^-1^min^-1^ | Rate of p38 Phosphorylation | Passos *et al* 2010 |
| kp38dphos | 6 min^-1^ | Rate of p38 Dephosphorylation | Passos *et al* 2010 |
| kROSgen | 1.5 min^-1^ | Rate of ROS Generation by p38 | Passos *et al* 2010 |
| kIR | 80 min^-1^ | Rate of IR Generation | New rate |
| kROS | 50 min^-1^ | Constant rate of ROS production | Measured |
| kdROS | 5 min^-1^ | ROS degradation Rate | Measured |
| kdam1 | 0.000006 #^-1^min^-1^ | Rate of simple damage production by ROS | New rate |
| kdam2 | 0.000006 #^-1^min^-1^ | Rate of complex damage production by ROS | New rate |
| kdku1 | 0.5 min^-1^ | Rate of ku dissociation from a simple break | Recalculated from Dolan *et al* 2013 |
| kdku2 | 0.5 min^-1^ | Rate of ku dissociation from a complex break | Recalculated from Dolan *et al* 2013 |
| kdku3 | 5 min^-1^ | Rate of oxidised ku dissociation from a simple break | Recalculated from Dolan *et al* 2013 |
| kdku4 | 5 min^-1^ | Rate of oxidised ku dissociation from a complex break | Recalculated from Dolan *et al* 2013 |
| kdnapk1 | 0.033 #^-1^min^-1^ | Rate of DNAPK simple break complex formation | Recalculated from Dolan *et al* 2013 |
| kdnapk2 | 0.0017 #^-1^min^-1^ | Rate of DNAPK complex break complex formation | Recalculated from Dolan *et al* 2013 |
| kddnapk1 | 0.02 min^-1^ | Rate of DNAPK simple break complex dissociation | Recalculated from Dolan *et al* 2013 |
| kddnapk2 | 0.02 min^-1^ | Rate of DNAPK complex break complex dissociation | Recalculated from Dolan *et al* 2013 |
| kdnapkphos1 | 0.28 min^-1^ | Rate of DNAPK phosphorylation in a DNAPK complex of a simple break | Dolan *et al* 2013 |
| kdnapkphos2 | 0.28 min^-1^ | Rate of DNAPK phosphorylation in a DNAPK complex of a complex break | Dolan *et al* 2013 |
| kliIV1 | 0.00071 #^-1^min^-1^ | Rate of LigaseIV binding to a simple break complex | Recalculated from Dolan *et al* 2013 |
| kliIV2 | 0.00046 #^-1^min^-1^ | Rate of LigaseIV binding to a complex break complex | Recalculated from Dolan *et al* 2013 |
| kdliIV1 | 0.0001 min^-1^ | Rate of LigaseIV dissociation from a simple break complex | Recalculated from Dolan *et al* 2013 |
| kdliIV2 | 0.0001 min^-1^ | Rate of LigaseIV dissociation from a complex break complex | Recalculated from Dolan *et al* 2013 |
| kfixIV1 | 0.0285 min^-1^ | Rate of DNA repair by LigaseIV of a simple break | Dolan *et al* 2013 |
| kfixIV2 | 0.0285 min^-1^ | Rate of DNA repair by LigaseIV of a complex break | Dolan *et al* 2013 |
| kPARP1 | 0.000077 #^-1^min^-1^ | Rate of PARP binding to simple break | Recalculated from Dolan *et al* 2013 |
| kPARP2 | 0.000065 #^-1^min^-1^ | Rate of PARP binding to complex break | Recalculated from Dolan *et al* 2013 |
| kdPARP1 | 0.02 min^-1^ | Rate of PARP dissociation from a simple break | Recalculated from Dolan *et al* 2013 |
| kdPARP2 | 0.02 min^-1^ | Rate of PARP dissociation from a complex break | Recalculated from Dolan *et al* 2013 |
| kliIII1 | 0.0015 #^-1^min^-1^ | Rate of LigaseIII binding to a simple break | Recalculated from Dolan *et al* 2013 |
| kliIII2 | 0.00024 #^-1^min^-1^ | Rate of LigaseIII binding to a complex break | Recalculated from Dolan *et al* 2013 |
| kdliIII1 | 0.0001 min^-1^ | Rate of LigaseIII dissociation from a simple break | Recalculated from Dolan *et al* 2013 |
| kdliIII2 | 0.0001 min^-1^ | Rate of LigaseIII dissociation from a complex break | Recalculated from Dolan *et al* 2013 |
| kfixIII1 | 0.0006 min^-1^ | Rate of DNA accurate repair by LigaseIII for a simple break | Dolan *et al* 2013 |
| kfixIII2 | 0.0006 min^-1^ | Rate of DNA accurate repair by LigaseIII for a complex break | Dolan *et al* 2013 |
| kfixIII3 | 0.0009 min^-1^ | Rate of DNA inaccurate repair by LigaseIII for a simple break | Dolan *et al* 2013 |
| kfixIII4 | 0.0009 min^-1^ | Rate of DNA inaccurate repair by LigaseIII for a complex break | Dolan *et al* 2013 |
| kATMact | 0.0012 min^-1^ | Rate of ATM Activation | New rate |
| kATMinact | 0.03 min^-1^ | Rate of ATM Inactivation | New rate |
| kh2axp1 | 0.1 #^-1^min^-1^ | Phosphorylation of h2ax via ATM | New rate |
| kh2axp2 | 0.5 min^-1^ | Phosphorylation of h2ax via DNA-PKcs | Dolan *et al* 2013 |
| kh2axu | 0.01 min^-1^ | Dephosphorylation of h2ax | Dolan *et al* 2013 |
| kh2axfoc | 0.5 min^-1^ | Creation of damage foci | Dolan *et al* 2013 |
| kfocback | 0.03 min^-1^ | Resolution of damage foci | Dolan *et al* 2013 |
| kh2axfull | 0.1 #^-1^min^-1^ | Creation of complete damage foci | Dolan *et al* 2013 |
| kfocfin | 0.03 min^-1^ | Resolution of complete damage foci | Dolan *et al* 2013 |
| kox | 0.000025 #^-1^min^-1^ | Rate of oxidisation of Ku | New rate |
| kred | 0.002 min^-1^ | Rate of reduction of Ku | New rate |
| ksen | 100 min^-1^ | Activation of senescent state | New rate |
